# Supplementary material for: Spatially and temporally probing distinctive glycerophospholipid alterations in Alzheimer’s disease mouse brain via high-resolution ion mobility-enabled sn-position resolved lipidomics
Source: Nat Commun. 2024 Jul 24;15:6252. doi: 10.1038/s41467-024-50299-9 (PMC11269705; doi:10.1038/s41467-024-50299-9)
Supplement: Supplementary file 1 — Supplementary Information [file 41467_2024_50299_MOESM1_ESM.pdf]

Supplementary information for

Spatially and temporally probing distinctive glycerophospholipid alterations in Alzheimer's disease mouse brain via high-resolution ion mobility-enabled *sn*-position resolved lipidomics

Shuling Xu[1, 9], Zhijun Zhu[2, 9], Daniel G. Delafield[2], Michael J. Rigby[3, 4, 5], Gaoyuan Lu[1], Megan Braun[3, 4, 5], Luigi Puglielli[3, 4, 6] & Lingjun Li[1, 2, 7, 8 *]

[1]School of Pharmacy, University of Wisconsin-Madison, Madison, WI 53705, USA

[2]Department of Chemistry, University of Wisconsin-Madison, Madison, WI 53706, USA

[3]Department of Medicine, University of Wisconsin-Madison, Madison, WI 53705, USA

[4]Waisman Center, University of Wisconsin-Madison, Madison, WI 53705, USA

[5]Neuroscience Training Program, University of Wisconsin-Madison, Madison, WI 53705, USA

[6]Geriatric Research Education Clinical Center, Veterans Affairs Medical Center, Madison, WI 53705, USA

[7]Lachman Institute for Pharmaceutical Development, School of Pharmacy, University of Wisconsin-Madison, Madison, WI 53705, USA

[8]Wisconsin Center for NanoBioSystems, School of Pharmacy, University of Wisconsin- Madison, Madison, WI 53705, USA

[9]These authors contributed equally: Shuling Xu, Zhijun Zhu.

*Corresponding author: Lingjun Li

Email: lingjun.li@wisc.edu

Mailing address: 777 Highland Ave, Madison, WI 53705, United States

Tel: +1 608-265-8491, Fax: +1 608-262-5345

Table of Content

List of Supplementary Notes, Figures, Tables, and Methods.

Supplementary Notes 1-3.

Supplementary Figure 1: Drift spectra of a series GP *sn*-position isomers.

Supplementary Figure 2: MS spectrum of GPs and the drift spectra of sodiated GPs by HRdm.

Supplementary Figure 3: Drift spectrum of PS 16:0_18:1 *sn*-isomer pairs in negative ion mode.

Supplementary Figure 4: Representative drift spectra of *sn*-position isomer mixtures with different ratios.

Supplementary Figure 5: Comparison of the quantification results using ion abundance in drift spectrum with PLA2 digestion method.

Supplementary Figure 6: Comparison of the drift spectra from isotope encoded lipid standards spiked in pure solvent and complex biological samples acquired with HRdm.

Supplementary Figure 7: Drift spectra of *sn*-isomer pure lyso-GPs obtained by PLA2 digestion of isopure lipid standards.

Supplementary Figure 8: The extracted ion mobilograms of GP *sn*-isomers acquired by TIMS.

Supplementary Figure 9: Ion mobility spectra of GP *sn*-isomers acquired by cyclic ion mobility spectrometry.

Supplementary Figure 10: IMS spectra of GP C═C bond position isomers.

Supplementary Figure 11: Energy-minimized structures and calculated theoretical CCS values of GPs.

Supplementary Figure 12: Assignment of lyso-GPs from drift spectra.

Supplementary Figure 13. The annotation workflow concluded from negative MS/MS spectra and drift spectra.

Supplementary Figure 14: Representative MS2 and drift spectra of GPs from mouse brain lipid extracts.

Supplementary Figure 15: Validations of GPs identified from brain tissues using available GP standards.

Supplementary Figure 16: Representative IM-MS heatmaps of GP *sn*-isomers identified from mouse brain.

Supplementary Figure 17: Schematic of the LC-IM-MS/MS strategy for GP identification.

Supplementary Figure 18: IM-MS heatmap and extracted drift spectra of PE 18:0_20:4 from different samples.

Supplementary Figure 19: Comparison of GPs identification number between reported strategy that 4D library-based match and rule-based refinement without HRdm (4D w/o HRdm) and our 4D lipidomics methods (4D w/ HRdm)

Supplementary Figure 20: Retention time prediction for GPs.

Supplementary Figure 21: Molecule percentage of GP classes in each brain region for WT and AD groups across clusters 1-5.

Supplementary Figure 22: Differential spatial alteration of *sn*-position GP isomers in 3 mouse brain regions between WT and AD.

Supplementary Figure 23: Principal component analysis (PCA) of AD and WT in three brain regions.

Supplementary Figure 24: Volcano plots showing significant GP changes in different brain regions of AD and aging mice.

Supplementary Figure 25: Compositional variations of GPs with C20:4 and DHA at *sn*-2 position among the AD and WT groups at 3 and 8 months in the cerebellum.

Supplementary Figure 26: Compositional variations of lyso-GPs and the ratio variations of representative GP/lyso-GP among the AD and WT groups at 3 and 8 months in the cerebellum.

Supplementary Figure 27: Compositional variations of GPs with C20:4 and DHA at *sn*-2 position among the AD and WT groups at 3 and 8 months in the cortex.

Supplementary Figure 28: Compositional variations of lyso-GPs and the ratio variations of representative GP/lyso-GP among the AD and WT groups at 3 and 8 months in the cortex.

Supplementary Figure 29: cPLA2 activities in mouse brain tissues from different genotypes and ages.

Supplementary Figure 30: Schematic representation of mouse brain dissection.

Supplementary Figure 31: The workflow for LC-high resolution-IM-MS/MS based 4D *sn*-resolved GP library construction.

Supplementary Table 1: The information on GP standards used in this study.

Supplementary Table 2: GP standards used for the comparison of quantitative performance of HRdm IM-MS with PLA2 digestion.

Supplementary Table 3: The list of the 35 most common fatty acids used for MDs calculation.

Supplementary Table 4: The list of optimized molecular descriptors calculated by CDK for CCS prediction.

Supplementary Table 5: The list of optimized molecular descriptors calculated by Mordred for CCS prediction.

Supplementary Table 6: The list of molecular descriptors with over 0.1% differences between *sn*-position isomers from Mordred.

Supplementary Table 7: The list of optimized 37 molecular descriptors from 253 molecular descriptors (including all 221 MDs from CDK and 32 Modred MDs which demonstrated > 0.1% difference between *sn*-position isomers) for building the CCS prediction model.

Supplementary Table 8: The list of optimized molecular descriptors calculated by CDK for retention time prediction.

Supplementary Methods.

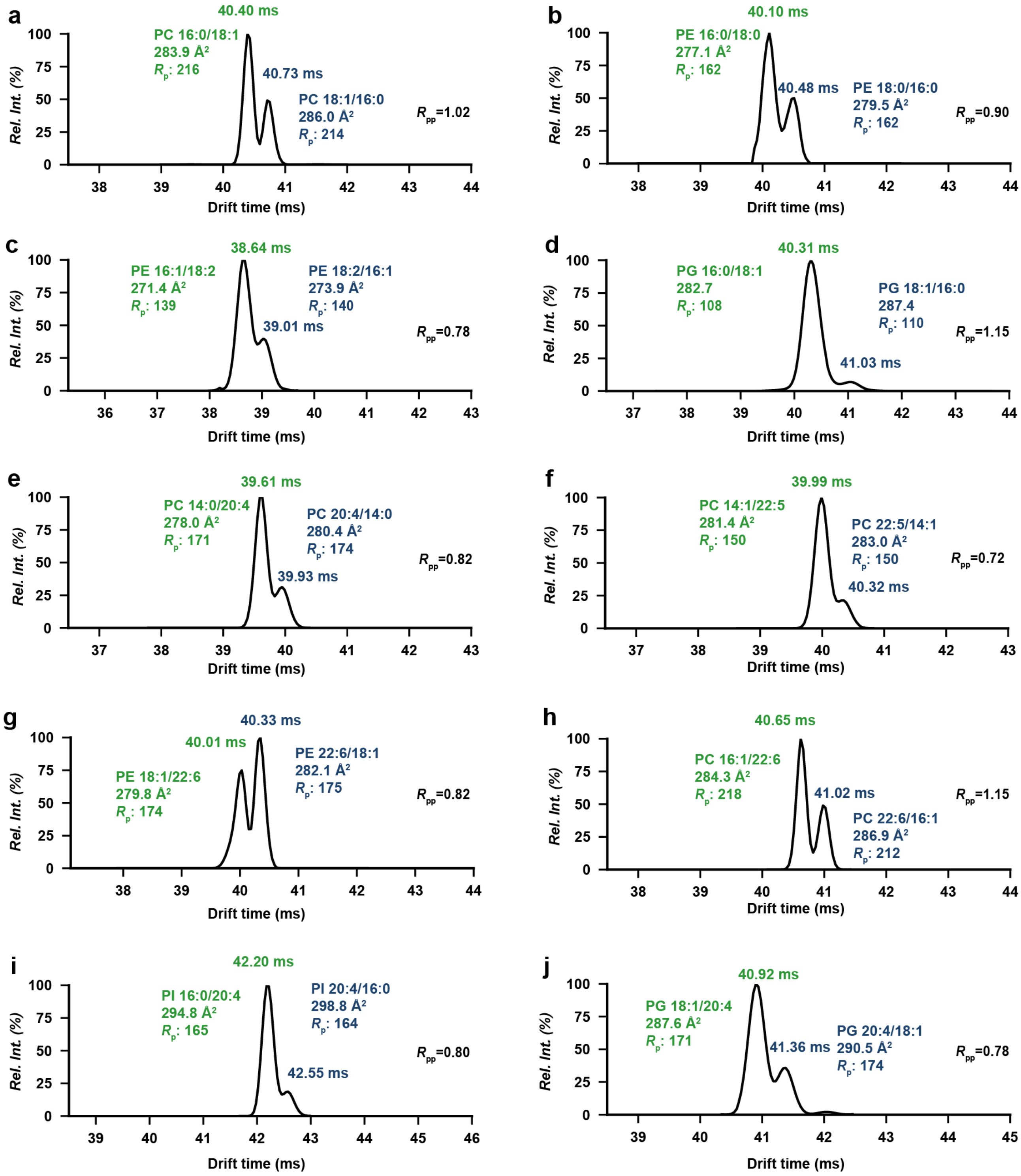

Supplementary Figure 1. Drift spectra of a series GP *sn*-position isomers. Drift spectra of PC 16:0_18:1 (**a**), PE 16:0_18:0 (**b**), PE 16:1_18:2 (**c**), PG 18:1_16:0 (**d**), PC 14:0_20:4 (**e**), PC 14:1_22:5 (**f**), PE 18:1_22:6 (**g**), PC 16:0_22:6 (**h**), PI 16:0_20:4 (**i**), and PG 18:1_22:4 (**j**). Source data are provided as a Source Data file.

Supplementary Note 1. Investigation of GP *sn*-position isomer separation in sodium adduction forms.

As metal ion adducts have previously been demonstrated to facilitate IM separation[1], we also evaluated the role of sodium adduction in GP *sn*-position isomer separation with HRdm. The *sn*-isomeric pair, PE 16:0/18:1(9Z) and PE 18:1(9Z)/16:0, were not separable in a mixture in their $[M+Na]^+$ forms but showed near baseline separation as $[M+2Na-H]^+$ ions (Supplementary Figure 2). These findings agree with previous literature that articulates multiple sodium adduction enhances the structural differences of isomeric small molecules[2]. However, because nonvolatile metal ions like Na^+ increase the propensity for instrumental contamination, we biased away from buffer systems and sample treatment that inspire sodium adduction. As a consequence, we observe relatively low abundance of GPs in the sodium-adducted forms, $[M+Na]^+$, and even less in $[M+2Na-H]^+$, which may be seen as an overall limitation when pursuing LC-based large-scale lipidomic analysis and quantitation. Our data so far have demonstrated HRdm provides excellent signal intensity and separation of GP *sn*-position isomers without adduction, indicating it is suitable and effective for comprehensive GP identification and quantification.

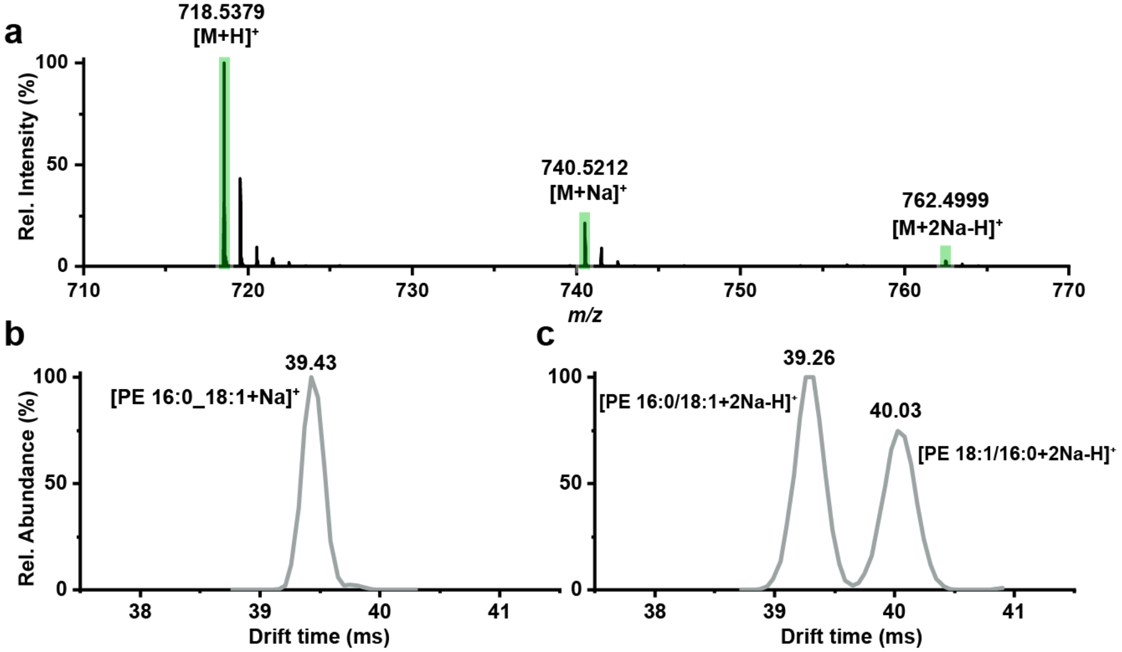

Supplementary Figure 2. MS spectrum of GPs and the drift spectra of sodiated GPs by HRdm. **a** Comparison of the abundance of PE 16:0_18:1(9Z) in $[M+H]^+$, $[M+Na]^+$, and $[M+2Na-H]^+$ in the MS spectrum. **b-c,** IMS spectra of PE 16:0_18:1(9Z) *sn*-position isomers in $[M+Na]^+$ (b) and $[M+2Na-H]^+$ (c). Source data are provided as a Source Data file.

Supplementary Note 2. The IM separation of GP *sn*-isomers in negative mode.

The IM separation of GP *sn*-isomers in negative mode was also investigated in the IM-MS spectrum. For example, the singly deprotonated ion form, $[M-H]^-$ of PS 16:0/18:1(9Z) and PS 18:1(9Z)/16:0 exhibited no differences in drift times and subsequently could not be resolved by HRdm IM (Supplementary Figure 3). Thus, positive mode was considered to be the most optimal polarity for HRdm analysis of GPs.

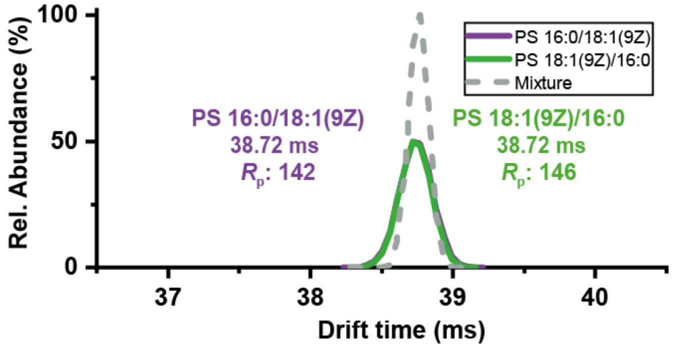

Supplementary Figure 3. Drift spectrum of PS 16:0_18:1 *sn*-isomer pairs in negative ion mode. Overlaid drift spectra of PS 16:0/18:1(9Z) (38.72 ± 0.02 ms) (Purple line), PS 18:1(9Z)/16:0 (38.72 ± 0.02 ms) (green line) (*n* = 3), and their equimolar mixture processed by HRdm in the negative ESI mode. Equal molar mixture is denoted with gray dashed lines. Source data are provided as a Source Data file.

Supplementary Note 3. Evaluation of quantification accuracy for *sn*-isomers using ion abundance in drift spectrum.

We assess the accuracy of quantification achievable in HRdm analyses, hypothesizing that ion abundance in the drift spectrum can be used to quantify GP *sn*-isomers. Quantification results from HRdm drift spectra demonstrated robust performance when evaluating PE 16:0_18:1 *sn*-isomers mixed at various ratios (Supplementary Figure 4). To further evaluate the quantitative accuracy, we tested *sn*-purities of non-isomer pure (may contain small amount of the other *sn*-isomer) GP commercial standards with HRdm IMS. Benchmarking with traditional phospholipase A2 (PLA2) digestion methods, which specifically cleaves GP fatty acyl chain at the *sn*-2 position, we found that *sn*-purities determined by the intensity ratios of released lyso-GP could well align with the HRdm results (Supplementary Table 2). Representing quantification data of non-isomer pure PE 16:0/18:1 commercial standard was shown as an example (Supplementary Figure 5).

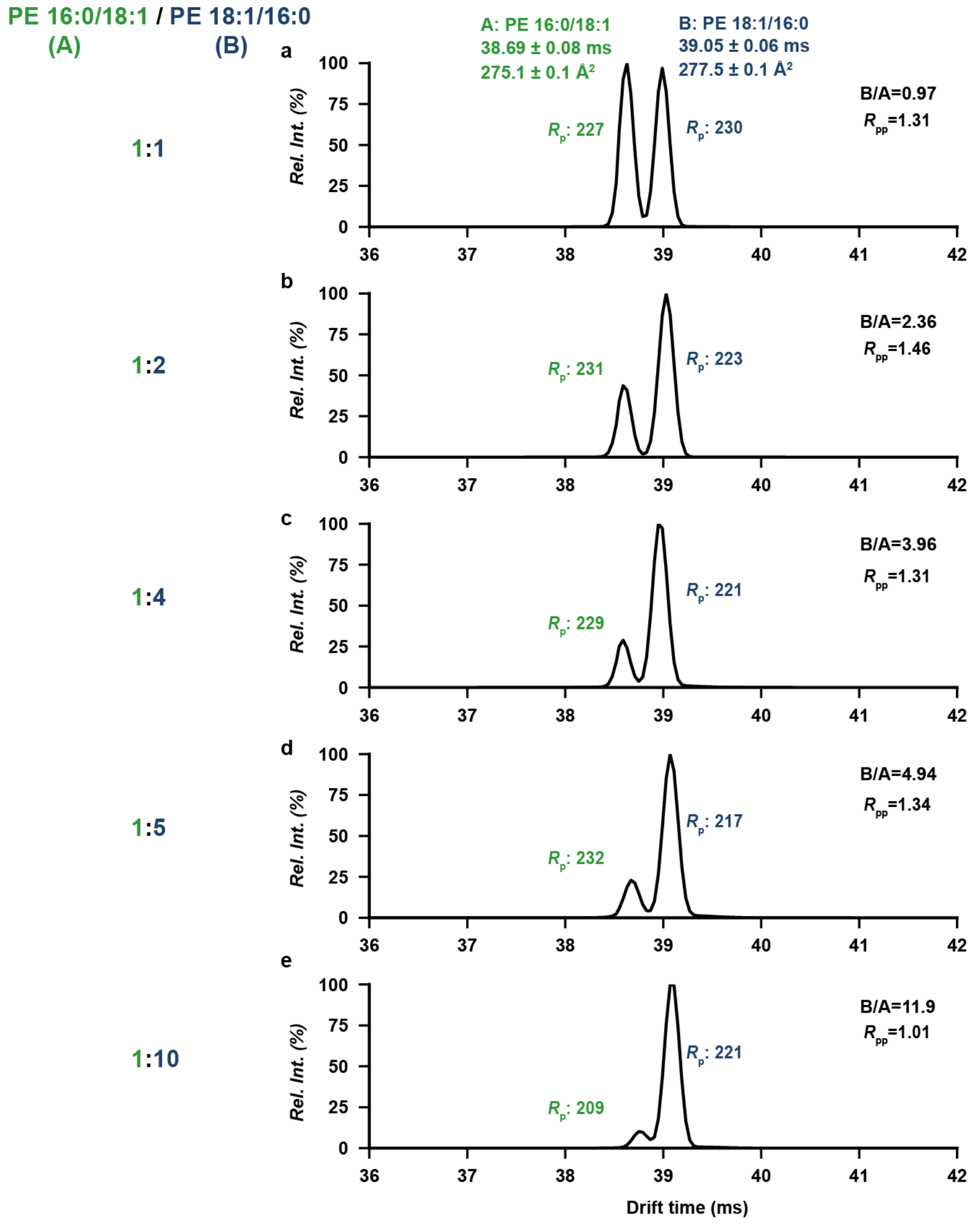

Supplementary Figure 4. Representative drift spectra of *sn*-position isomer mixtures at various ratios. PE 16:0/18:1 and PE 18:1/16:0 were mixed at molar ratios of 1:1 (**a**), 1:2 (**b**), 1:4 (**c**), 1:5 (**d**), and 1:10 (**e**). Source data are provided as a Source Data file.

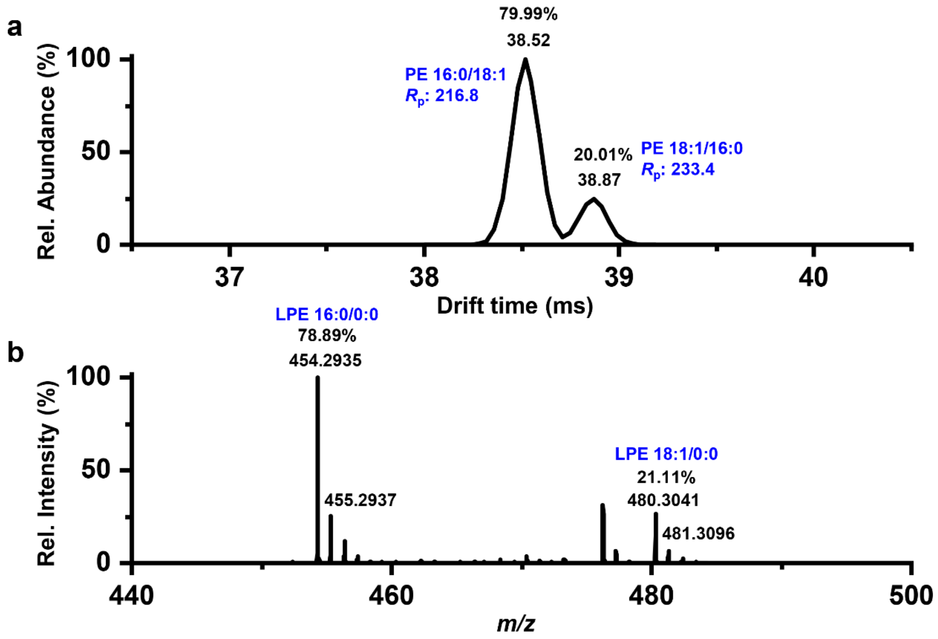

Supplementary Figure 5. Comparison of the quantification results using ion abundance in drift spectrum with PLA2 digestion method. a Drift spectrum of non-isopure PE 16:0/18:1 standard. **b** MS spectrum of lyso-PE from non-isopure PE 16:0/18:1 standard digested by PLA2. Source data are provided as a Source Data file.

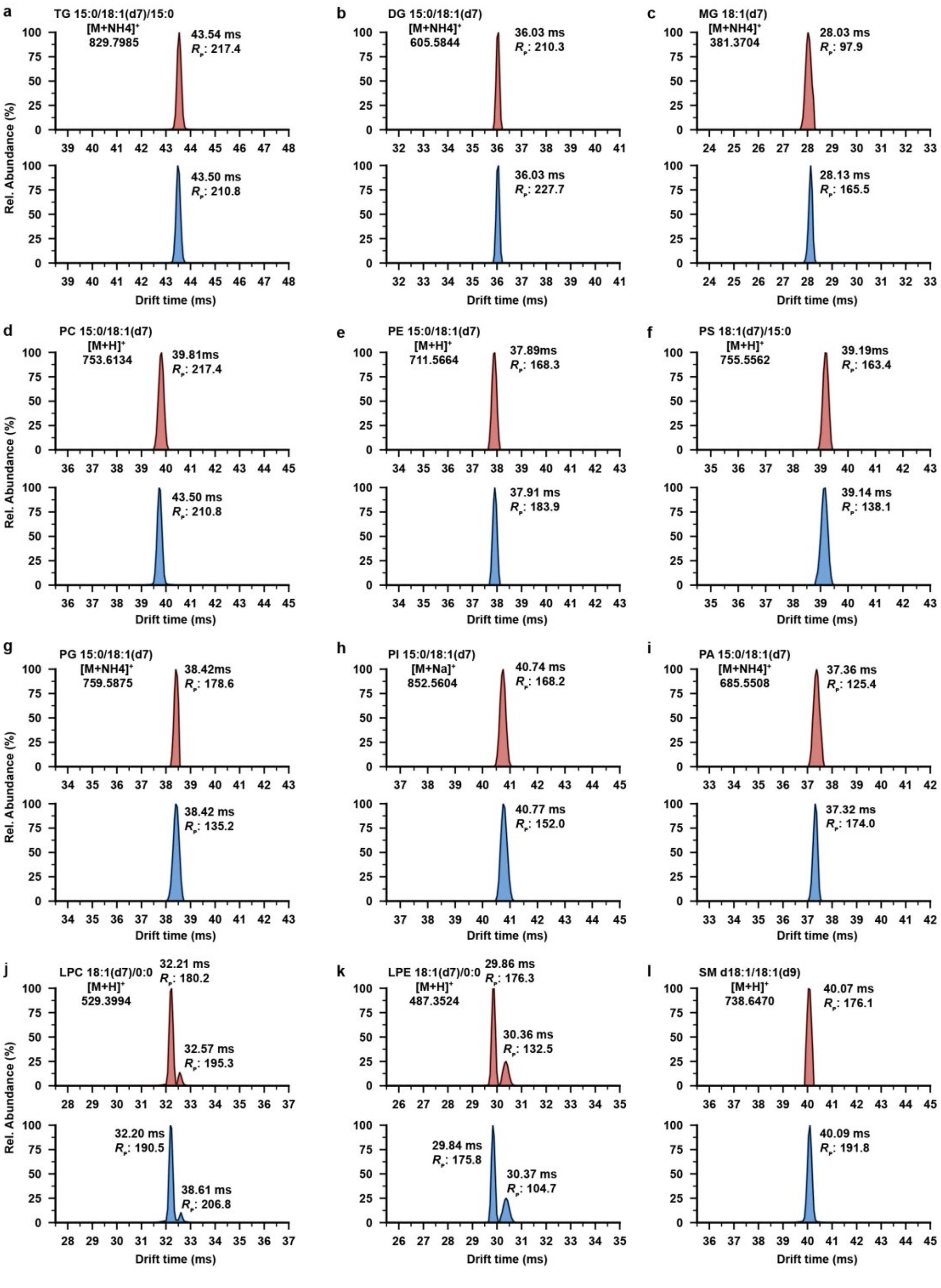

Supplementary Figure 6. Comparison of the drift spectra from isotope encoded lipid standards spiked in pure solvent (isopropanol) and complex biological samples (mouse brain tissue) acquired with HRdm. Drift spectra of TG 15:0/18:1(d7)/15:0 (**a**), DG 15:0/18:1(d7) (**b**), MG 18:1(d7) (**c**), PC 15:0/18:1(d7) (**d**), PE 15:0/18:1(d7) (**e**), PS 15:0/18:1(d7) (**f**), PG 15:0/18:1(d7) (**g**), PI 15:0/18:1(d7) (**h**), PA 15:0/18:1(d7) (**i**), LPC 18:1(d7) (**j**), LPE 18:1 (d7) (**k**), SM d18:1/18:1 (d9) (**l**) in moue brain sample (upper, red) and solvent (bottom, blue). Source data are provided as a Source Data file.

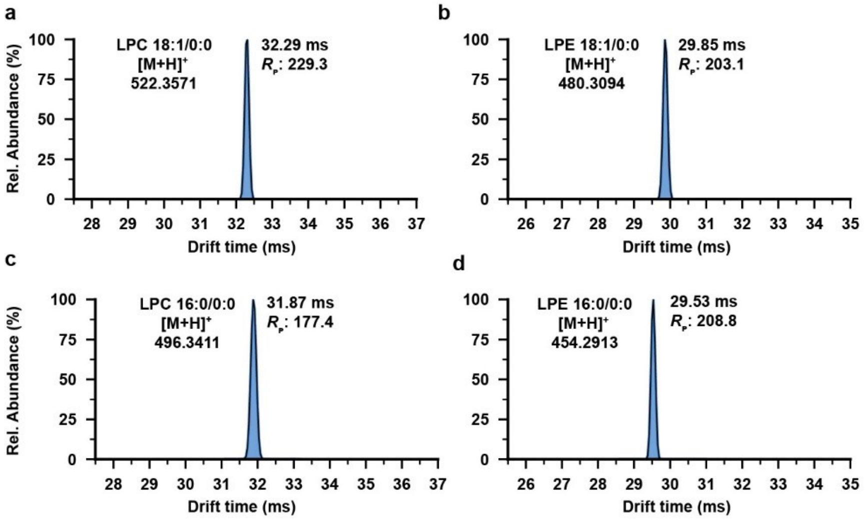

Supplementary Figure 7. Drift spectra of *sn*-isomer pure lyso-GPs obtained by PLA2 digestion of isopure lipid standards. Drift spectra of LPC 18:1/0:0 digested from PC 18:1(9Z)/16:0 (**a**), LPE 18:1/0:0 digested from PE 18:1(9Z)/16:0 (**b**), LPC 16:0 digested from PC 16:0/18:1(9Z) (**c**), LPE 16:0 digested from PE 16:0/18:1(9Z) (**d**). Source data are provided as a Source Data file.

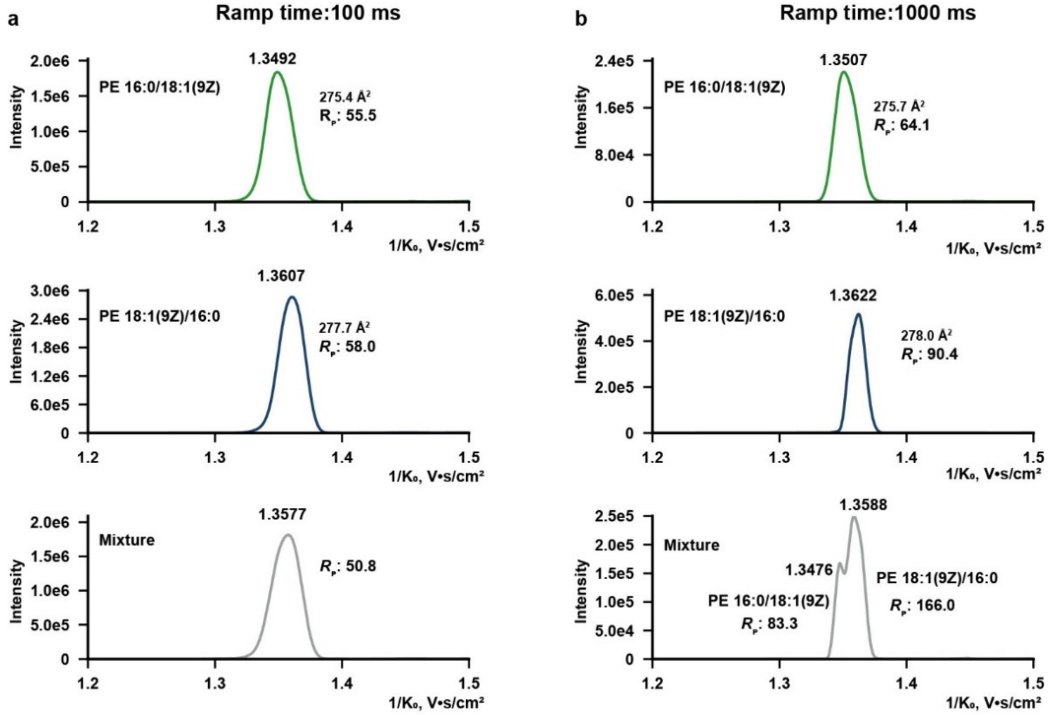

Supplementary Figure 8. The extracted ion mobilograms of GP *sn*-isomers acquired by TIMS. The extracted ion mobilograms of deprotonated PE 16:0/18:1(9Z) (green), PE 18:1(9Z)/16:0 (blue) and equimolar mixture of the *sn*-isomer pairs (dark) (at m/z 718.5381) acquired using 100 ms ramp time (**a**) and 1000 ms ramp time (**b**). Source data are provided as a Source Data file.

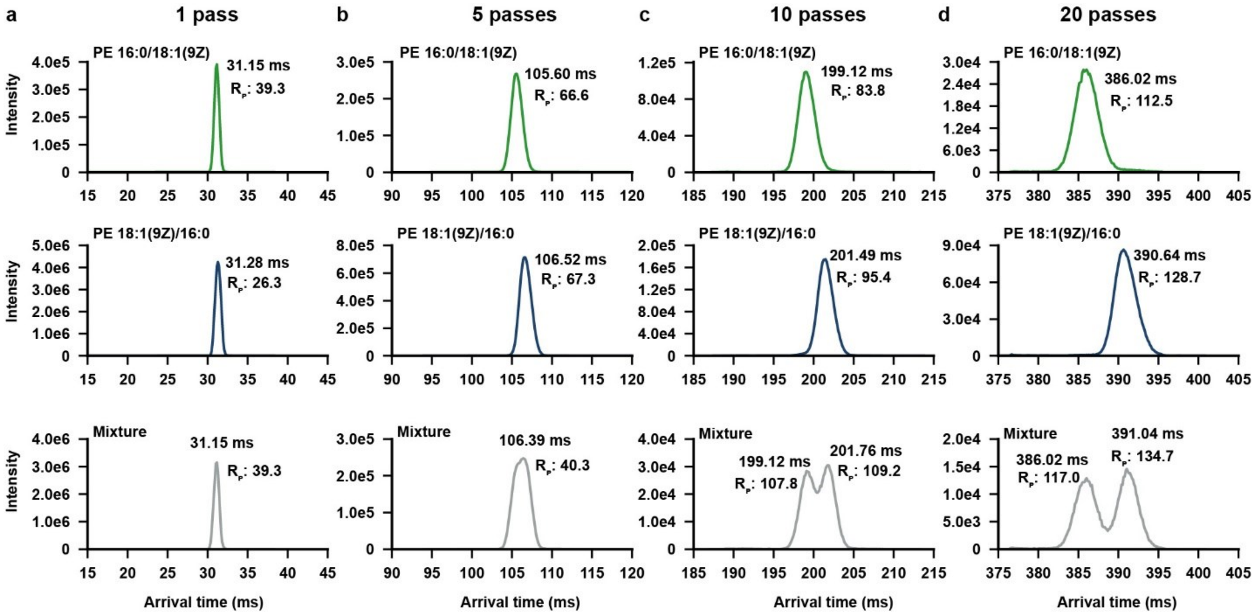

Supplementary Figure 9. Ion mobility spectra of GP *sn*-isomers acquired by cyclic ion mobility spectrometry. Ion mobility spectra of deprotonated PE 16:0/18:1(9Z) (green), PE 18:1(9Z)/16:0 (blue) and equimolar mixture of the *sn*-isomer pairs (dark) (at m/z 718.5381) after 1 pass (**a**), 5 passes (**b**), 10 passes (**c**), and 20 passes (**d**) in cIM device. Source data are provided as a Source Data file.

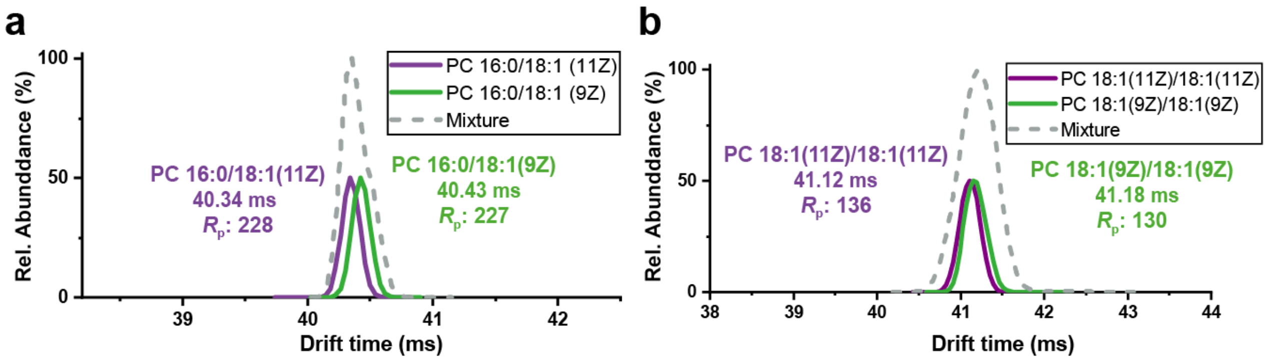

Supplementary Figure 10. IMS spectra of GP C═C bond position isomers. a Overlaid drift spectra of PC 16:0/18:1(9Z) (40.34 ± 0.03 ms), PC 16:0/18:1(11Z) (40.43 ± 0.02 ms), and their equimolar mixture processed by HRdm. **b** Overlaid drift spectra of PC 18:1(9Z)/18:1(9Z) (41.12 ± 0.01 ms), PC 18:1(11Z)/18:1(11Z) (41.18 ± 0.03 ms), and their equimolar mixture processed by HRdm (n = 3). Equal molar mixtures of respective GP C═C bond position isomers are denoted with gray dashed lines. Source data are provided as a Source Data file.

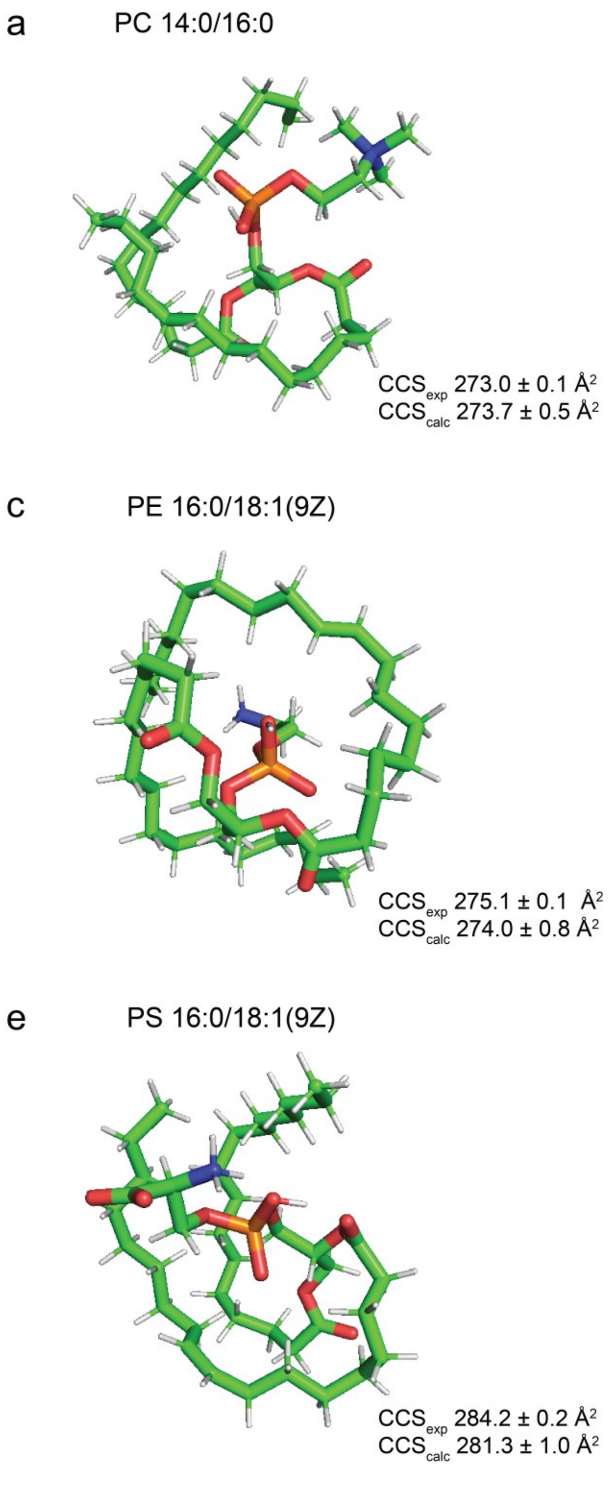

Supplementary Figure 11. Energy-minimized structures and calculated theoretical CCS values of GPs. Representative simulated structures of PC 14:0/16:0 (**a**), PC 16:0/14:0 (**b**), PE 16:0/18:1(9Z) (**c**), PE 18:1(9Z)/16:0 (**d**), PS 16:0/18:1(9Z) (**e**), and PS 18:1(9Z)/16:0 (**f**). Source data are provided as a Source Data file.

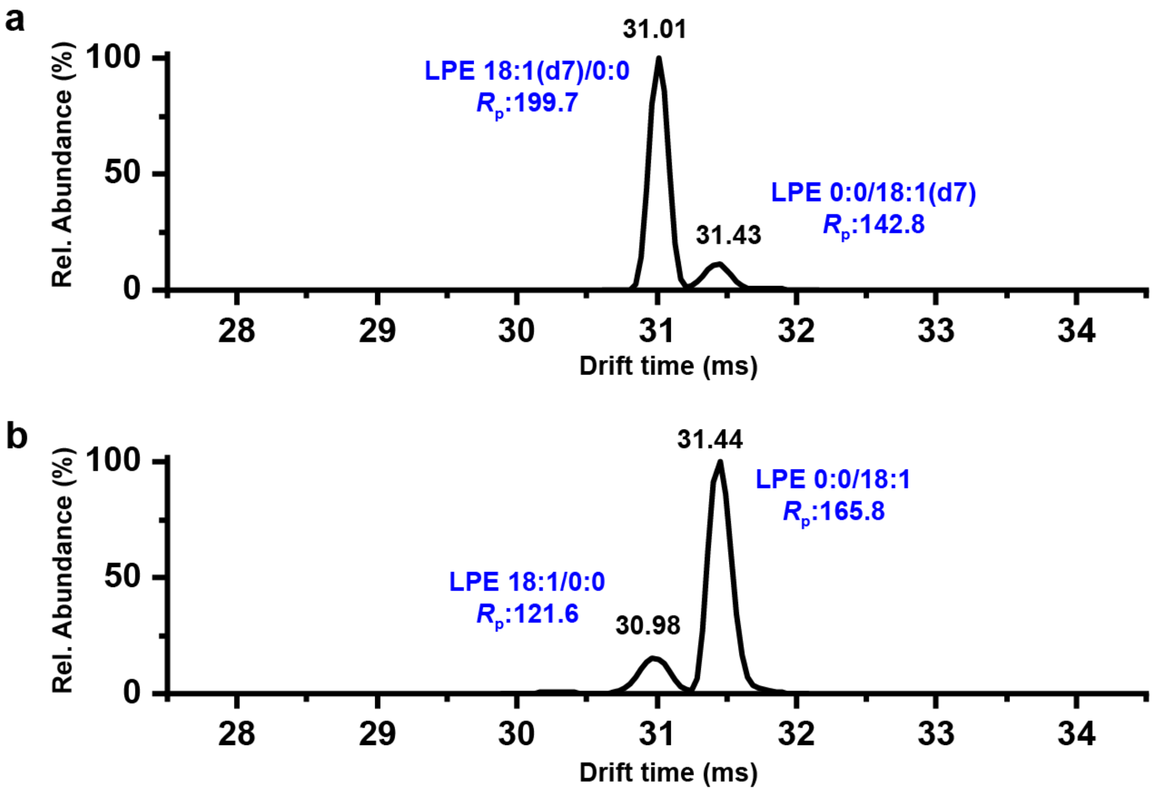

Supplementary Figure 12. Assignment of lyso-GPs from drift spectra. **a** IMS spectrum of LPE 18:1(d7)/0:0 standard. **b** IMS spectrum of LPE 18:1 mixture in brain samples. As LPE 18:1(d7)/0:0 was not 100% pure, a small amount of LPE 0:0/18:1(d7) was observed. The drift time and CCS of LPE 18:1 in biological samples were aligned well with stable isotope standards. The CCS values of LPE 18:1 *sn*-isomers indicated that lyso-GPs contained fatty acyl at *sn*-1 has the smaller CCS values. The rule was consistent with previous studies[3,4]. Source data are provided as a Source Data file.

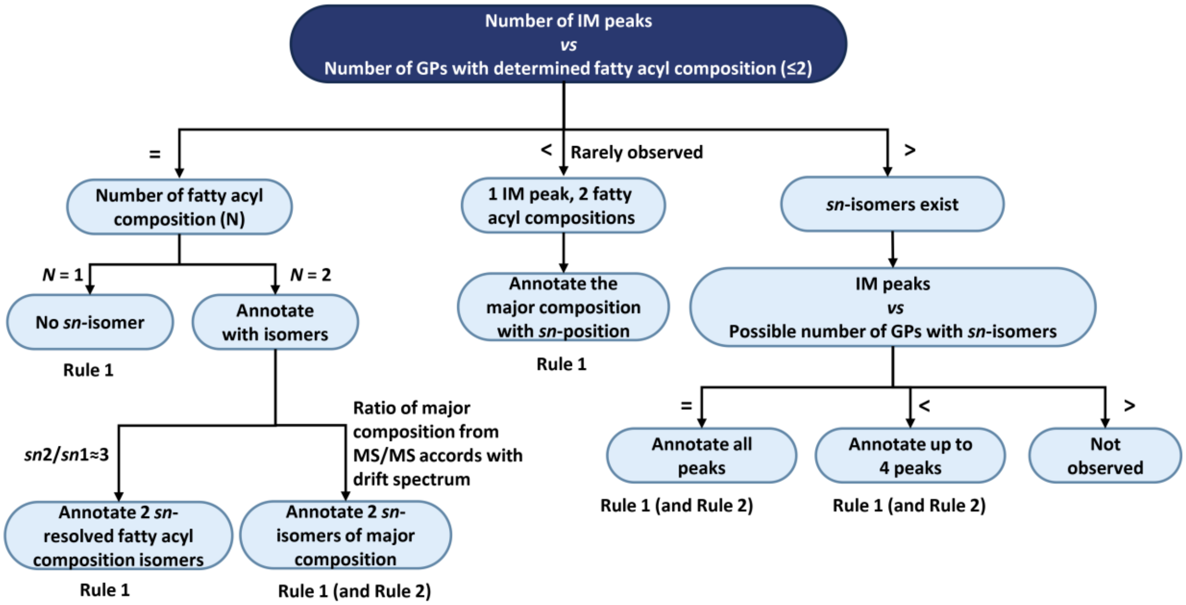

Supplementary Figure 13. The annotation workflow concluded from negative MS/MS spectra and drift spectra. IM peaks indicate genuine peaks after filtering out artificial peaks.

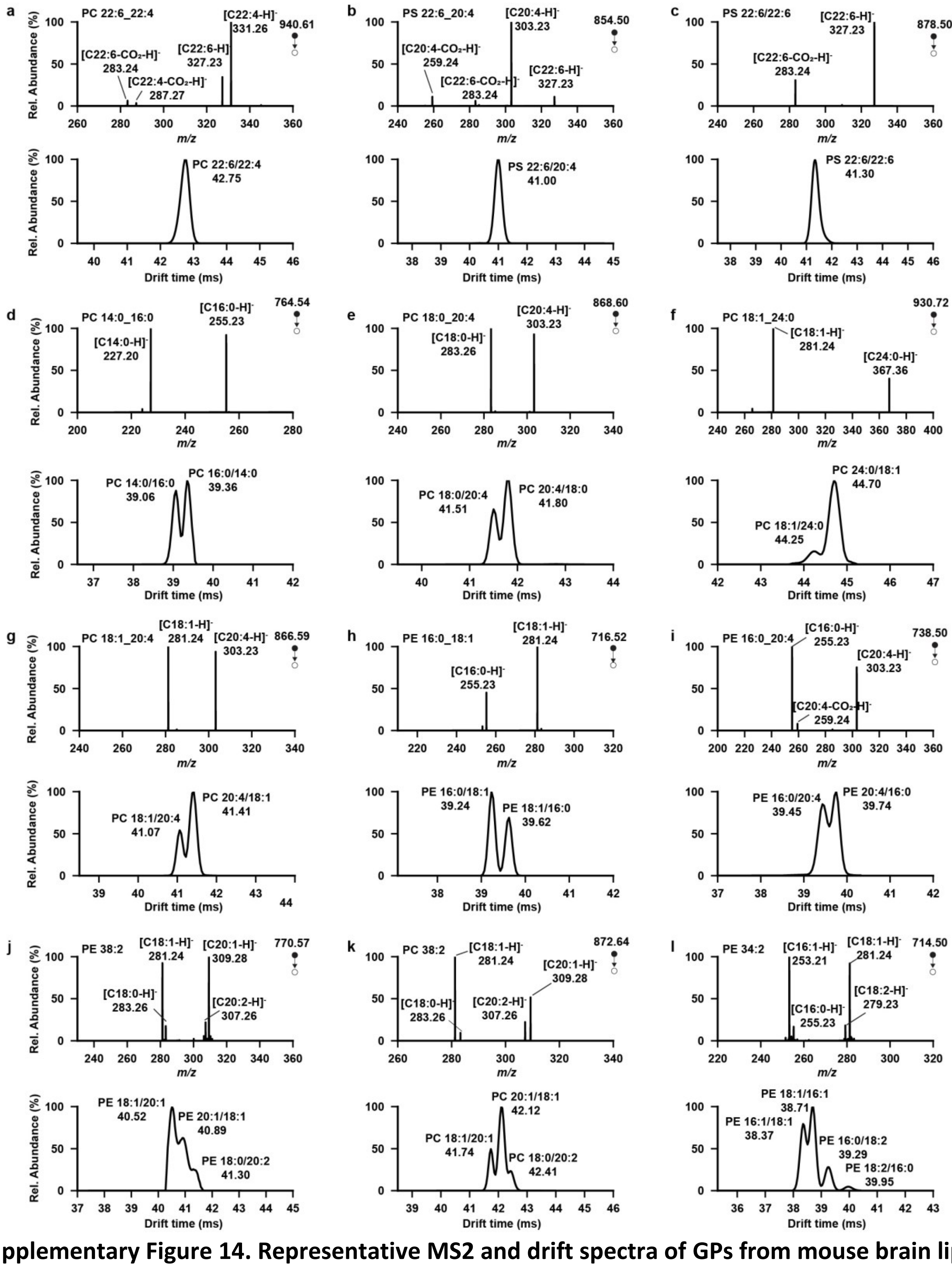

Supplementary Figure 14. Representative MS2 and drift spectra of GPs from mouse brain lipid extracts. MS2 and drift spectra of PC 22:6/22:4 (**a**), PS 22:6/20:4 (**b**), PS 22:6/22:6 (**c**), PC 14:0_16:0 (**d**), PC 18:0_20:4 (**e**), PC 18:1_24:0 (**f**), PC 18:1_20:4 (**g**), PE 16:0_18:1 (**h**), PE 16:0_20:4 (**i**), PE 38:2 (**j**), PC 38:2 (**k**), and PE 34:2 (**l**). Source data are provided as a Source Data file.

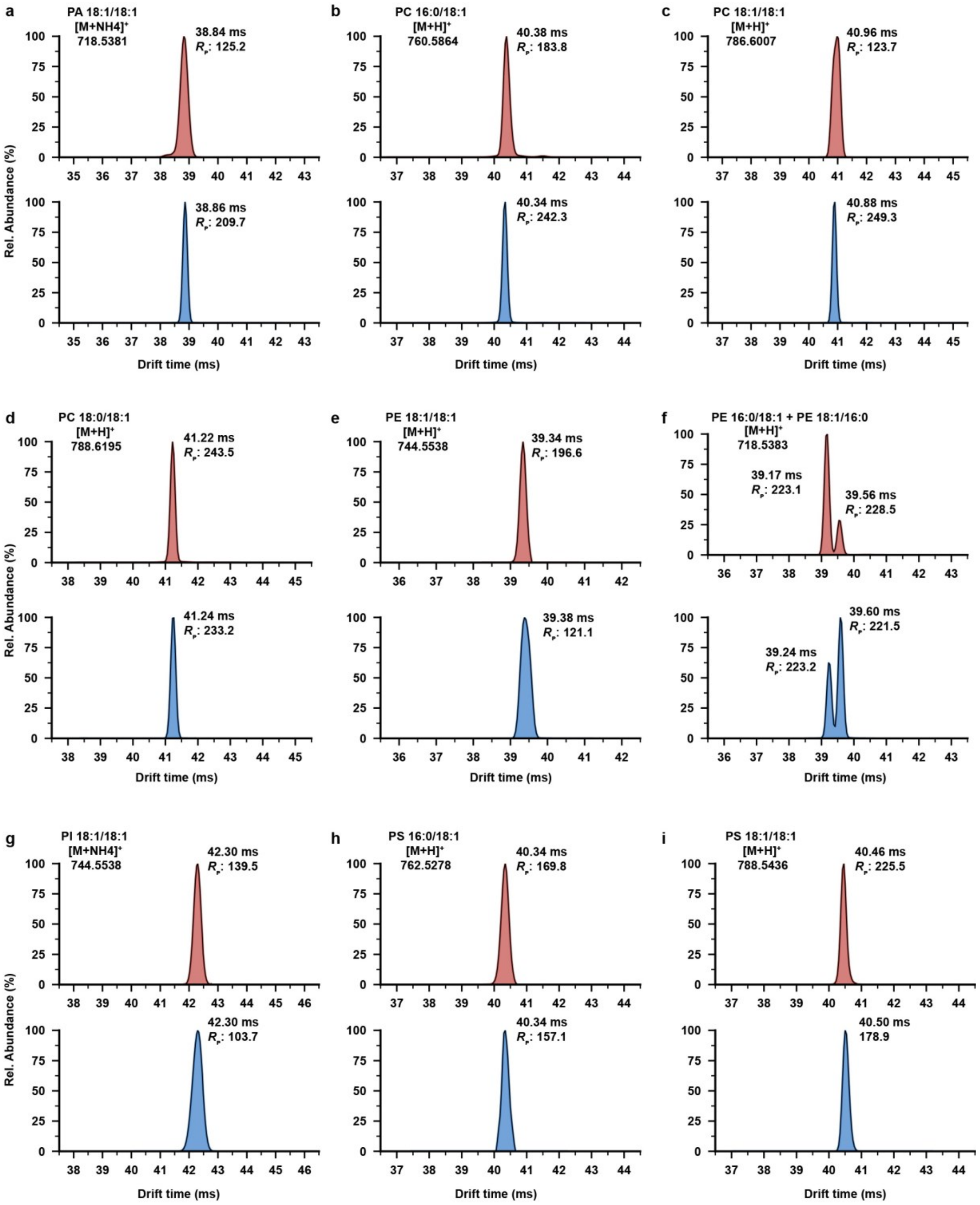

Supplementary Figure 15. Validations of GPs identified from brain tissues using available GP standards. Drift time alignment of GPs in brain samples (red) and standards (blue) measured in the same batch, PA 18:1/18:1(**a**), PC 16:0/18:1 (**b**), PC 18:1/18:1 (**c**), PE 18:1/18:1 (**d**), PE 16:0/18:1 (**e**), PE 18:1/16:0 (**f**), PI 18:1/18:1 (**g**), PS 16:0/18:1 (**h**), PS 18:1/18:1 (**i**). Source data are provided as a Source Data file.

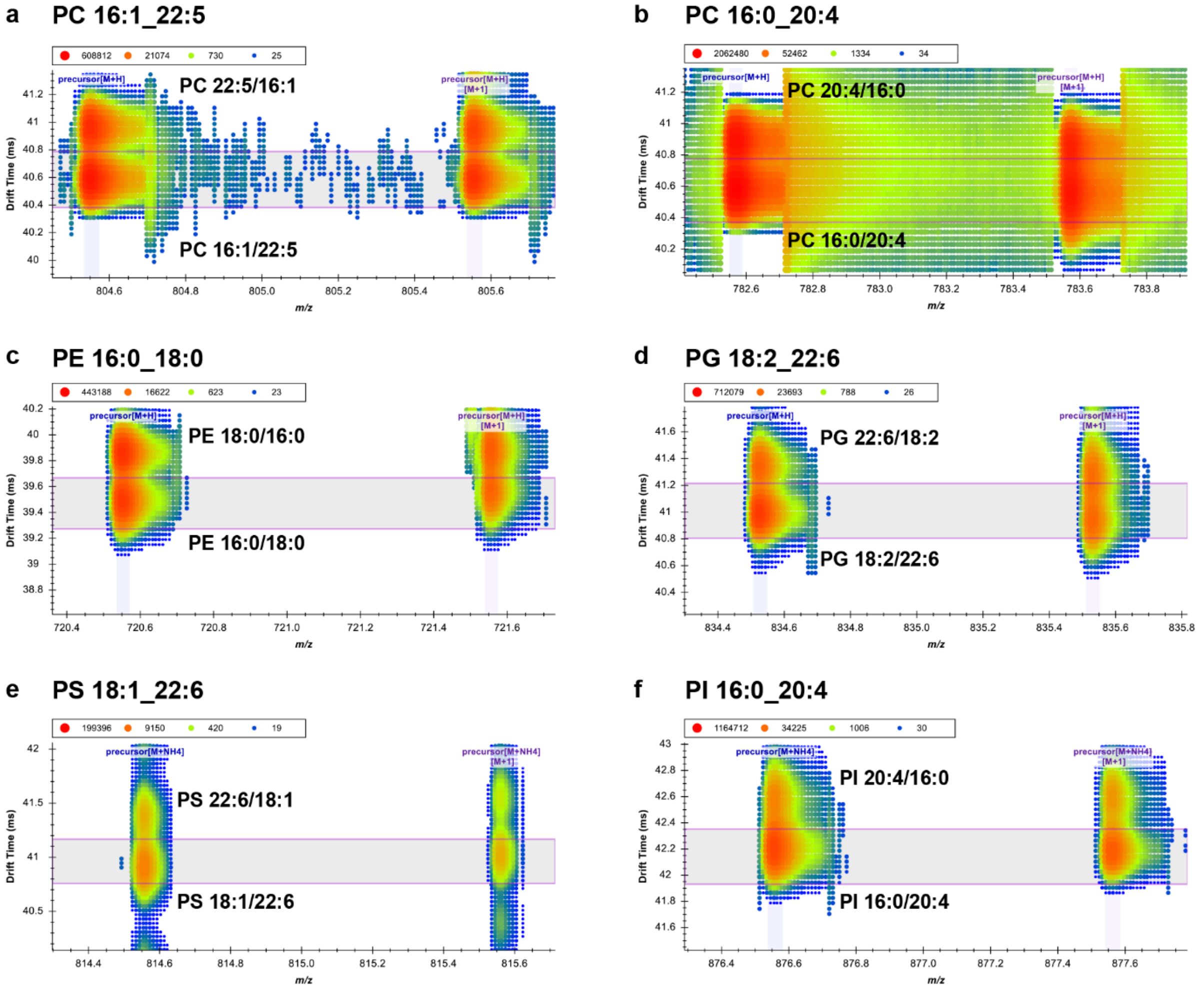

Supplementary Figure 16. Representative IM-MS heatmaps of GP *sn*-isomers identified from mouse brain. IM-MS heatmaps of PC 16:1_22:5 (**a**), PC 16:0_20:4 (**b**), PE 16:0_18:0 (**c**), PG 18:2_22:6 (**d**), PS 18:1_22:6 (**e**), PI 16:0_20:4 (**f**). Dashed grey lines marked the extracted mobility range windows of the isomers with smaller CCS values. Source data are provided as a Source Data file.

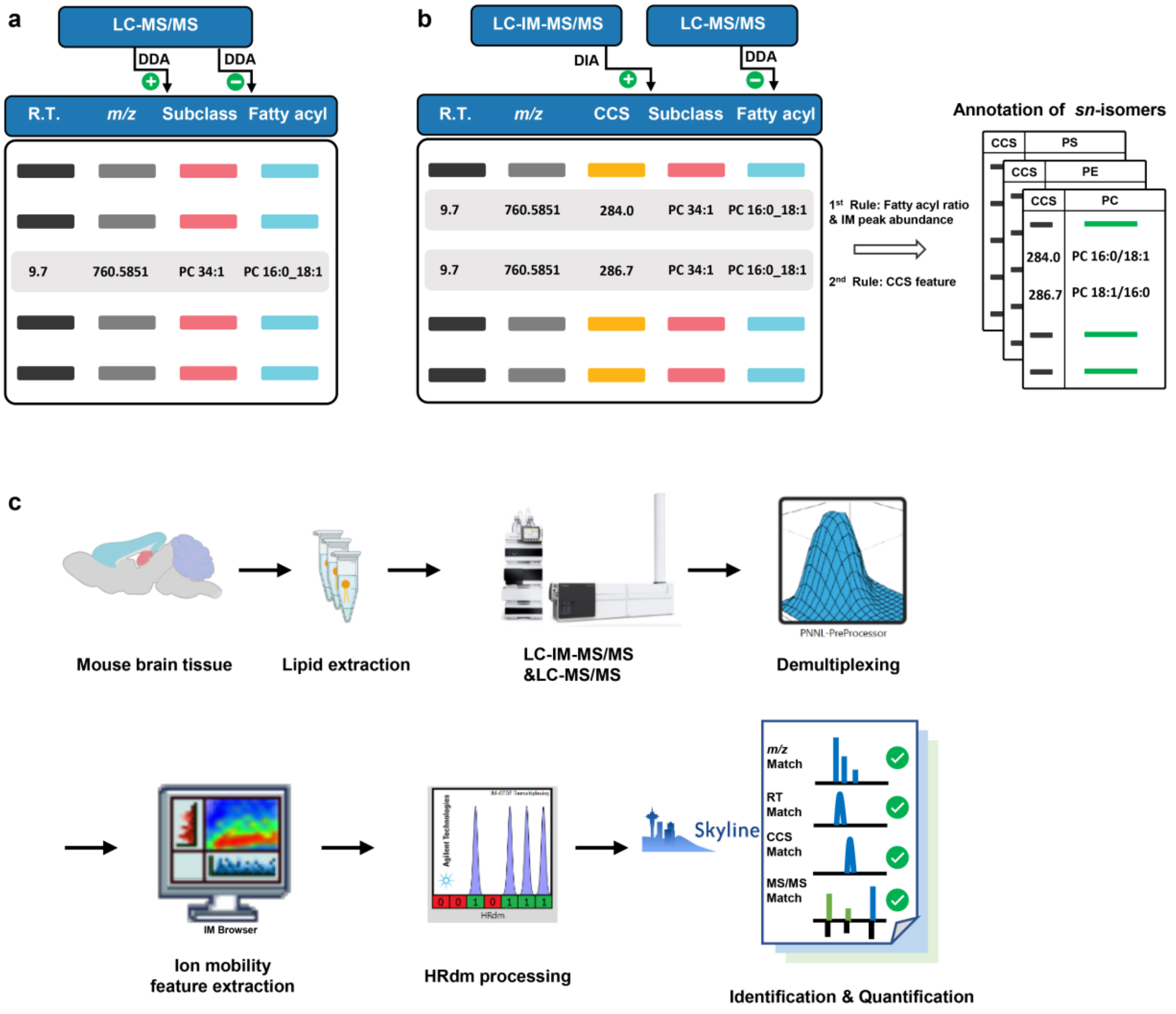

Supplementary Figure 17. Schematic of the LC-IM-MS/MS strategy for GP identification. a The outline of data dependent LC–MS/MS acquisition for lipid identification at fatty acyl composition level using QTOF-only mode without IM measurement. **b** The outline of integrated data independent LC-IM-MS/MS and data dependent LC-MS/MS acquisition for lipid identification at *sn*-position resolved level. **c** The overall workflow for GP analysis in biological samples including detailed data processing analysis. Supplementary Figure 17c was created with BioRender.com, released under a Creative Commons Attribution-NonCommercial-NoDerivs 4.0 International license.

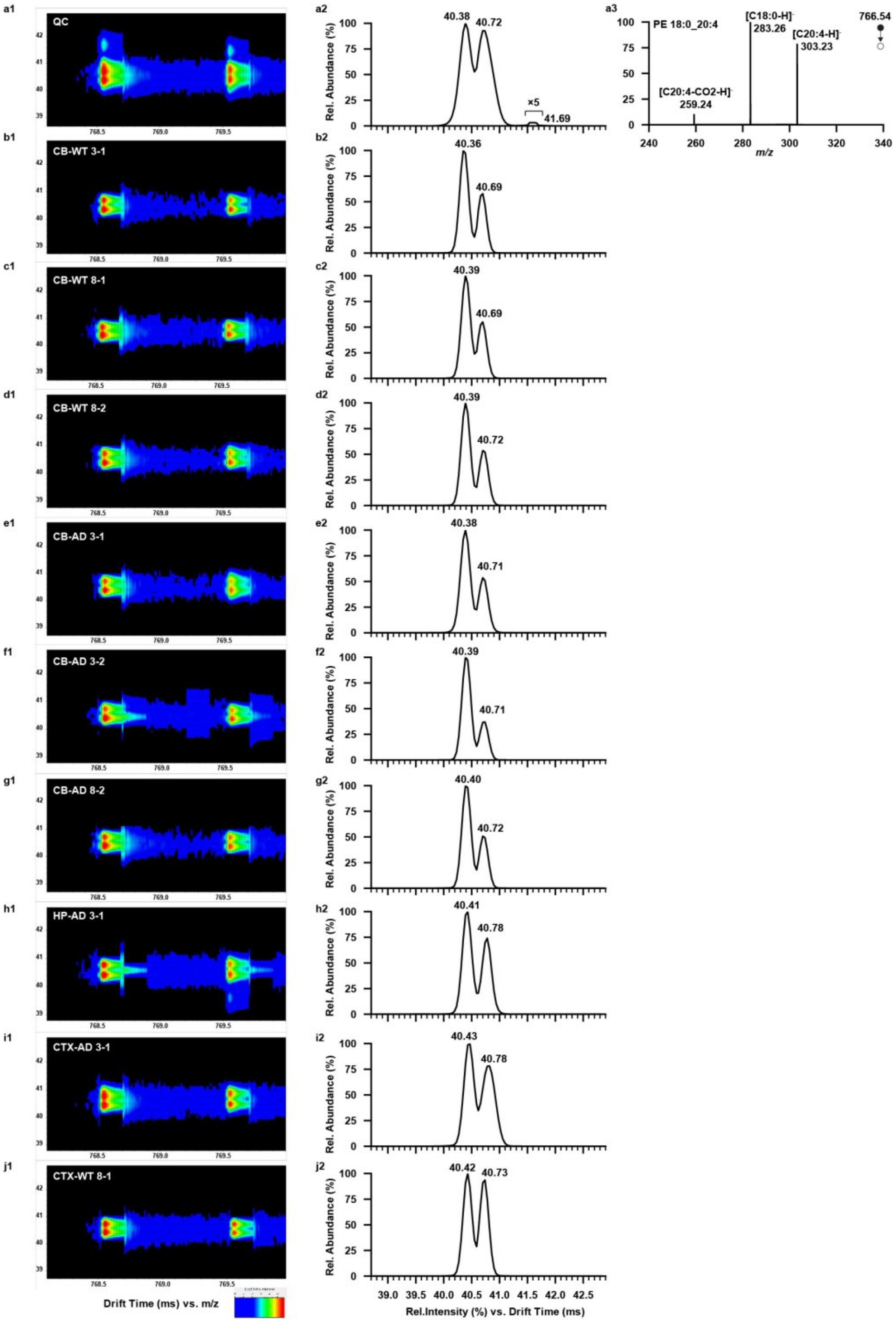

Supplementary Figure 18. IM-MS heatmap and extracted drift spectra of PE 18:0_20:4 from different samples. IM-MS heatmap and extracted drift spectra of PE 18:0_20:4 from QC sample (**a1**, **a2**), CB-WT 3-1 (**b1**, **b2**), CB-WT 8-1 (**c1**, **c2**), CB-WT 8-2 (**d1**, **d2**), CB-AD 3-1 (**e1**, **e2**), CB-AD 3-2 (**f1**, **f2**), CB-AD-8-2 (**g1**, **g2**), HP-AD 3-1 (**h1**, **h2**), CTX AD 3-1 (**i1**, **i2**), and CTX-WT 8-1(**j1**, **j2**), respectively. **a3** Negative MS2 Spectrum of PE 18:0_20:4 from QC sample. Source data are provided as a Source Data file.

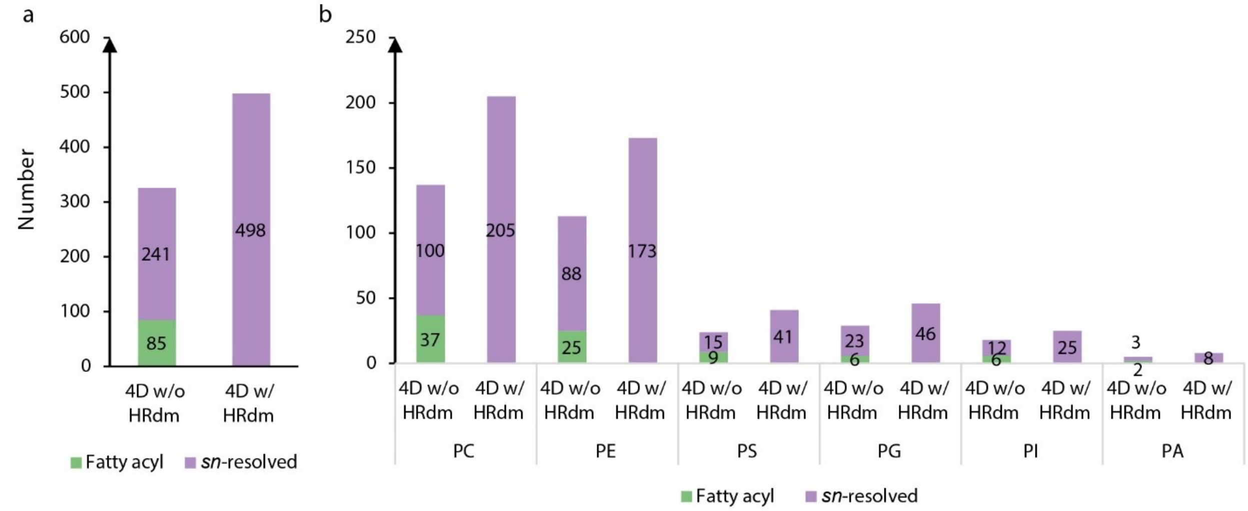

Supplementary Figure 19. Comparison of GPs identification number between reported strategy using 4D library-based match and rule-based refinement without HRdm (4D w/o HRdm) and our 4D lipidomics strategy (4D w/ HRdm). a Comparison of total identification number of GPs between 4D w/o HRdm and 4D w/ HRdm strategies. **b** Comparison of identification number of each GP class between 4D w/o HRdm and 4D w/ HRdm strategies. Source data are provided as a Source Data file.

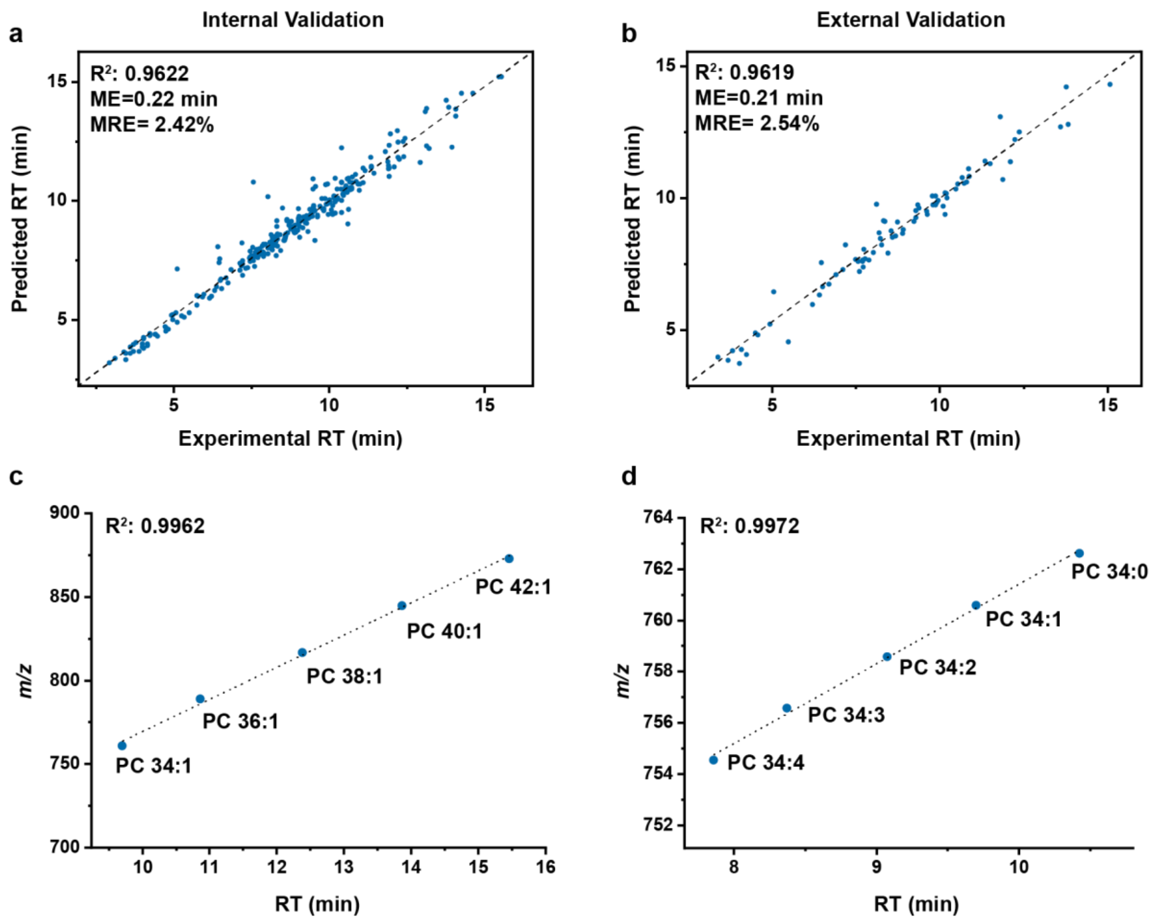

Supplementary Figure 20. Retention time prediction for GPs. a The internal validation of the machine learning algorithm predicted retention time of GPs (n = 335) using optimized MDs selected from CDK. **b** The prediction performance was evaluated by external validation (n = 84). **c-d,** Retention times of PCs identified in mouse brain differed in either carbon numbers (c) or double bond numbers (d). Source data are provided as a Source Data file.

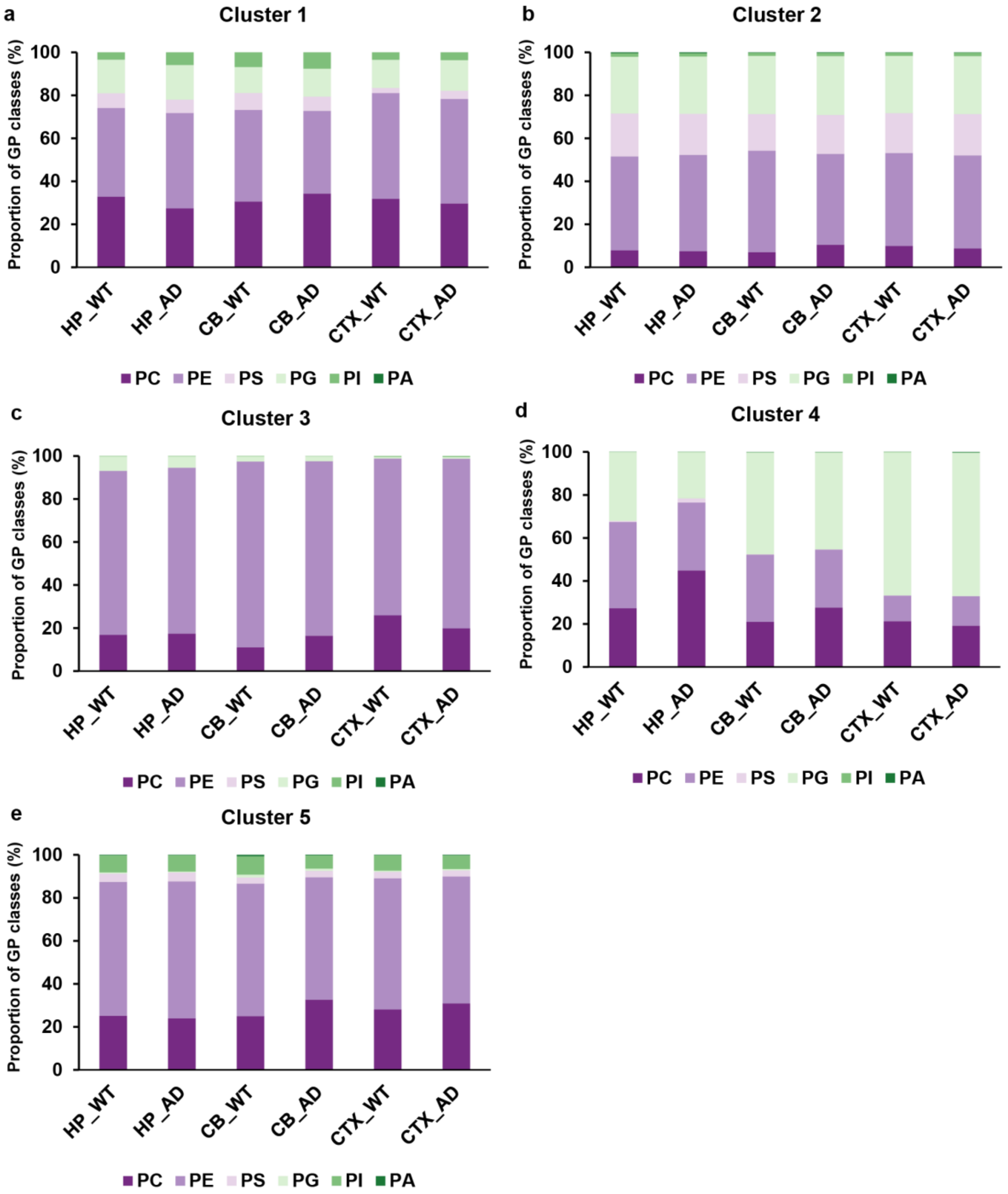

Supplementary Figure 21. Molecule percentage of GP classes in each brain region for WT and AD groups across clusters 1-5. Proportions of GP classes (%) in cluster 1 (**a**), cluster 2 (**b**), cluster 3 (**c**), cluster 4 (**d**), and cluster 5 (**e**) from Figure 4f are shown. The average concentration of each GP, calculated from biological replicates within the same group, was used. Source data are provided as a Source Data file.

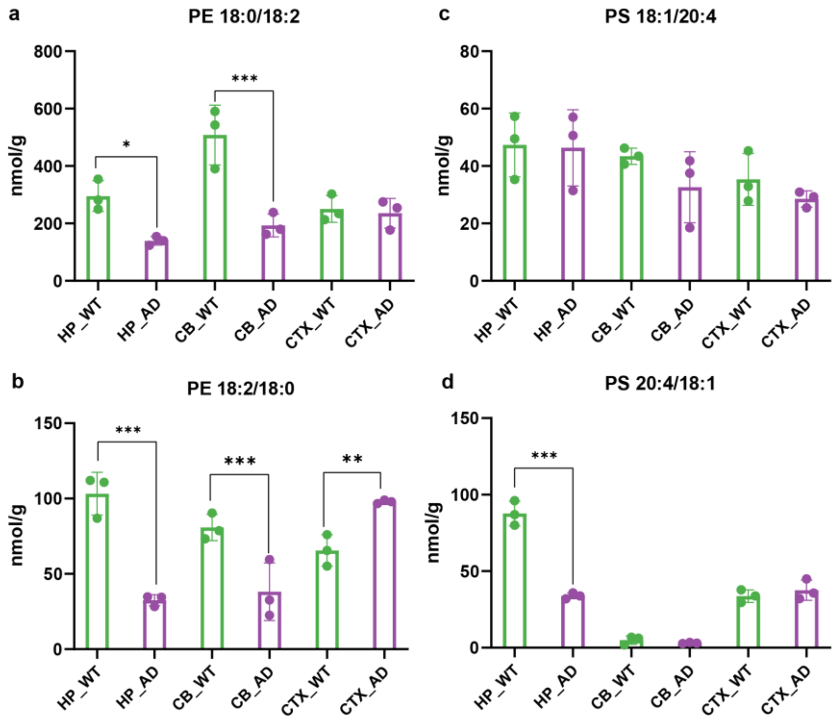

Supplementary Figure 22. Differential spatial alteration of *sn*-position GP isomers in 3 mouse brain regions between WT and AD. Concentration of PE 18:0/18:2 (p = 0.0105 for HP, 0.0001 for CB) (**a**), PE 18:2/18:0 (p = 1.9E-5 for HP, 4.2E-4 for CB, 0.0020 for CTX) (**b**), PS 18:1/20:4 (**c**), PS 20:4/18:1 (p = 1.0E-8 for HP) (**d**). Data are presented as mean values +/−SD (n = 3, *p < 0.05, **p < 0.01, ***p < 0.001 (one-way ANOVA with correction for multiple comparisons using the two-stage linear step-up procedure of Benjamin, Krieger and Yekutieli at a 0.05 FDR). Source data are provided as a Source Data file.

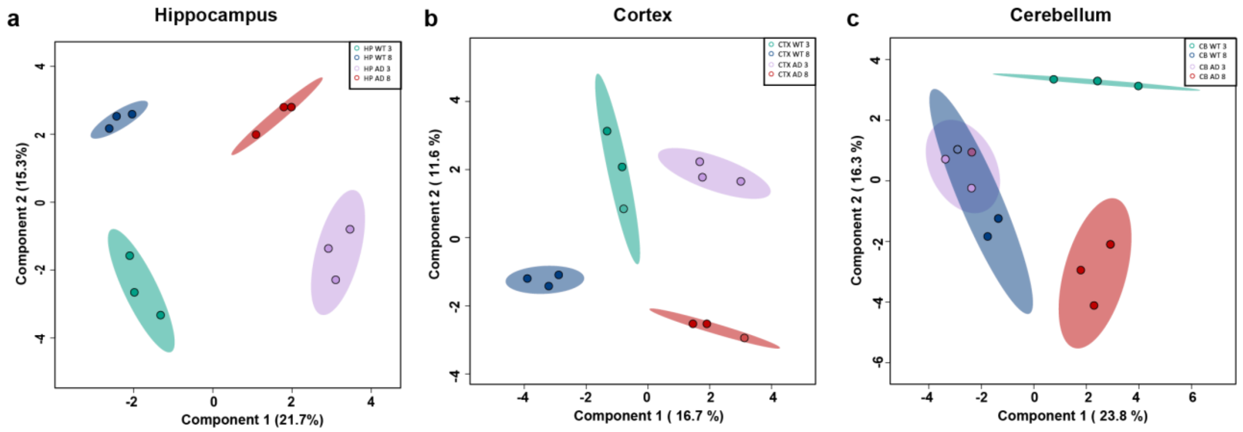

Supplementary Figure 23. Principal component analysis (PCA) of AD and WT mouse brain in three brain regions. PCA of hippocampus (**a**), cortex (**b**), and cerebellum (**c**). Source data are provided as a Source Data file.

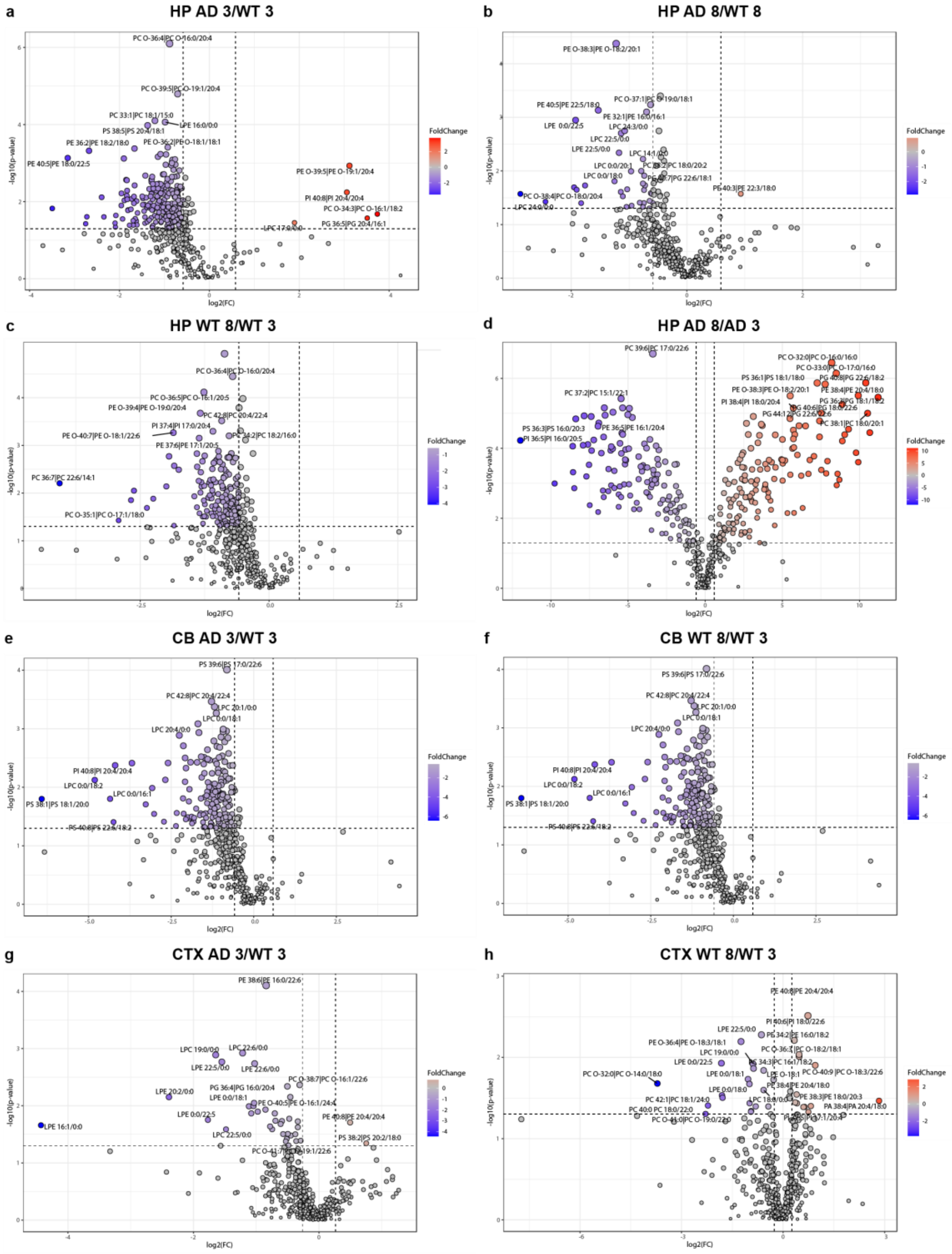

Supplementary Figure 24. Volcano plots showing significant GP changes in different brain regions of AD and aging mice. GPs with significant changes ($p < 0.05$, fold change >1.5) are highlighted in the volcano plots, including AD 3 vs WT3 of hippocampus (**a**), AD 8 vs WT 8 of hippocampus (**b**), WT 8 vs WT 3 of hippocampus (**c**), AD 8 vs AD 3 of hippocampus (**d**), AD 3 vs WT3 of cerebellum (**e**), WT 8 vs WT 3 of cerebellum (**f**), AD 3 vs WT3 of cortex (**g**), WT 8 vs WT 3 of cortex (**h**).

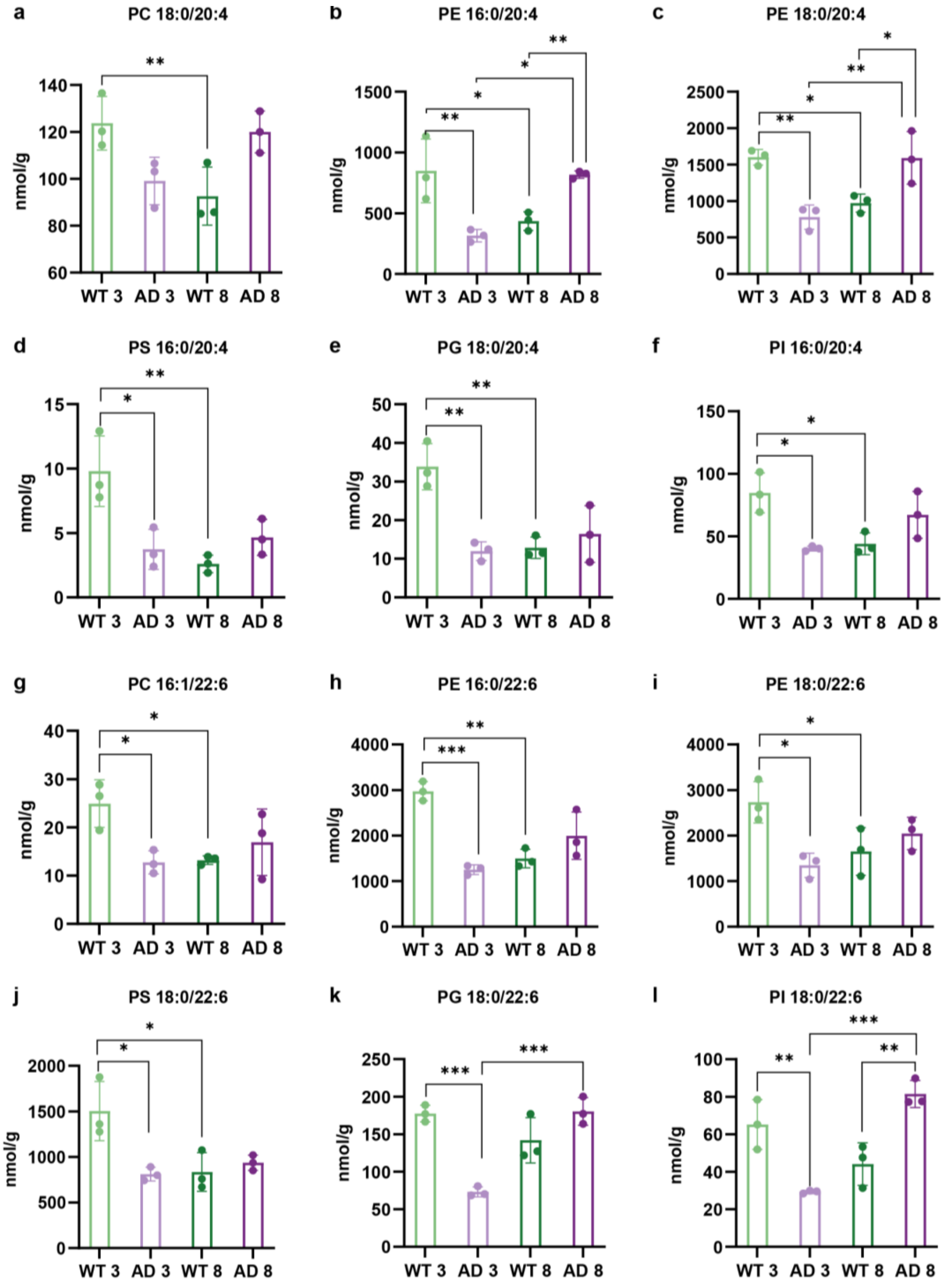

Supplementary Figure 25. Compositional variations of GPs with C20:4 and DHA at *sn*-2 position among the AD and WT groups at 3 and 8 months in the cerebellum. Concentrations of PC 18:0/20:4 (*p* (WT 3 vs. WT 8) = 0.0318) (**a**), PE 16:0/20:4 (*p* (WT 3 vs. AD 3) = 0.0069, *p* (WT 3 vs. WT 8) = 0.0277, *p* (AD 3 vs. AD 8)= 0.0098, *p* (WT 8 vs. AD 8)= 0.0403) (**b**), PE 18:0/20:4 (*p* (WT 3 vs. AD 3) = 0.0070, *p* (WT 3 vs. WT 8) = 0.0298, *p* (AD 3 vs. AD 8)= 0.0074, *p* (WT 8 vs. AD 8)= 0.0320) (**c**), PS 16:0/20:4 (*p* (WT 3 vs. AD 3) = 0.0122, *p* (WT 3 vs. WT 8) =

0.0045) (**d**), PG 18:0/20:4 (*p* (WT 3 vs. AD 3) = 0.0033, *p* (WT 3 vs. WT 8) = 0.0043) (**e**), PI 16:0/20:4 (*p* (WT 3 vs. AD 3) = 0.0135, *p* (WT 3 vs. WT 8) = 0.0217) (**f**), PC 16:1/22:6 (*p* (WT 3 vs. AD 3) = 0.0399, *p* (WT 3 vs. WT 8) = 0.0480) (**g**), PE 16:0/22:6 (*p* (WT 3 vs. AD 3) = 0.0005, *p* (WT 3 vs. WT 8) = 0.0015) (**h**), PE 18:0/22:6 (*p* (WT 3 vs. AD 3) = 0.0142, *p* (WT 3 vs. WT 8) = 0.0490) (**i**), PS 18:0/22:6 (*p* (WT 3 vs. AD 3) = 0.0135, *p* (WT 3 vs. WT 8) = 0.0217) (j), PG 18:0/22:6 (*p* (WT 3 vs. AD 3) = 0.0006, *p* (AD 3 vs. AD 8) = 0.0005) (**k**), PI 18:0/22:6 (*p* (WT 3 vs. AD 3) = 0.0072, *p* (AD3 vs. AD 8) = 0.0007, *p* (WT 8 vs. AD 8) = 0.0056) (**l**) in cerebellum. Data are presented as mean values +/−SD ($n = 3$, *$p < 0.05$, **$p < 0.01$, ***$p < 0.001$ (one-way ANOVA, with correction for multiple comparisons at a 0.05 FDR)). Source data are provided as a Source Data file.

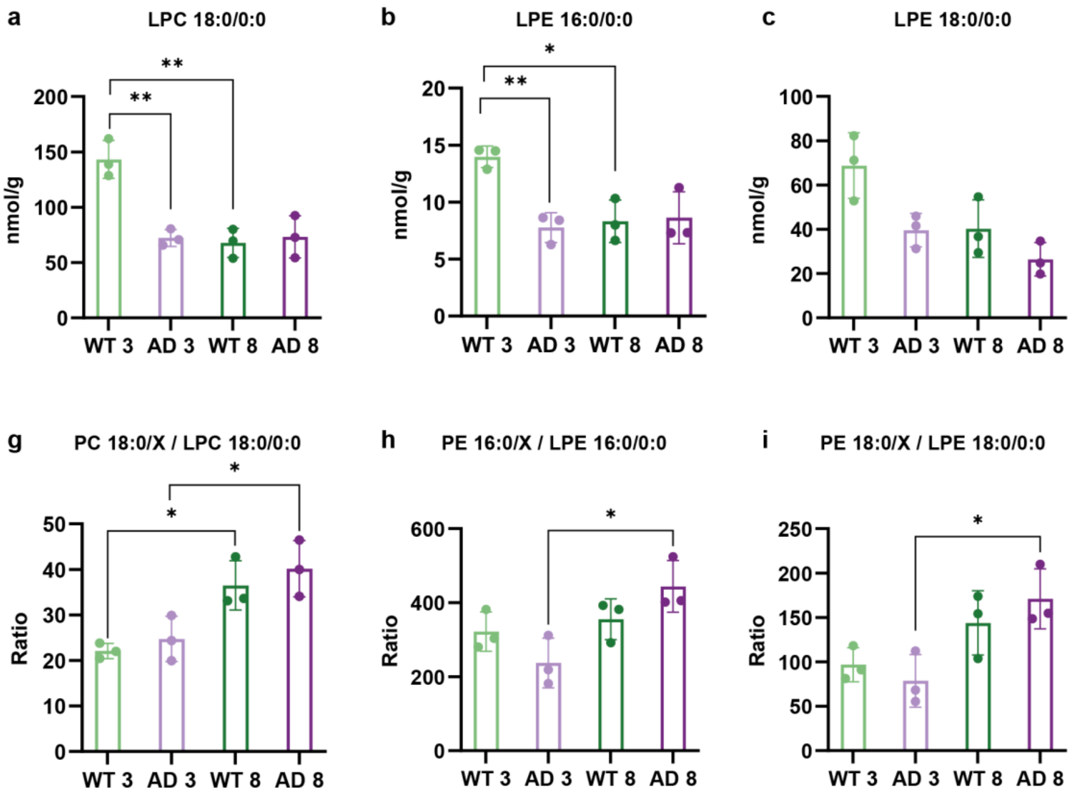

Supplementary Figure 26. Compositional variations of representative lyso-GPs and the ratio variations of representative GP/lyso-GP among the AD and WT groups at 3 and 8 months in the cerebellum. Concentrations of various lyso-GPs and ratios of corresponding GP/lyso-GP are presented: LPC 18:0/0:0 (*p* (WT 3 vs. AD 3) = 0.0017, *p* (WT 3 vs. WT 8) = 0.0012) (**a**), LPE 16:0/0:0 (*p* (WT 3 vs. AD 3) = 0.0083, *p* (WT 3 vs. WT 8) = 0.0140) (**b**), LPE 18:0/0:0 (**c**), PC 18:0/X / LPC 18:0/0:0 (*p* (WT 3 vs. WT 8) = 0.0281, *p* (AD 3 vs. AD 8) = 0.0197) (**d**), PE 16:0/X / LPE 16:0/0:0 (*p* (AD 3 vs. AD 8) = 0.0146) (**e**), PE 18:0/X / LPE 18:0/0:0 (*p* (AD 3 vs. AD 8) = 0.0243) (**f**). Data are presented as mean values +/−SD ($n = 3$, *$p < 0.05$, **$p < 0.01$, ***$p < 0.001$ (one-way ANOVA, with correction for multiple comparisons at a 0.05 FDR)). Source data are provided as a Source Data file.

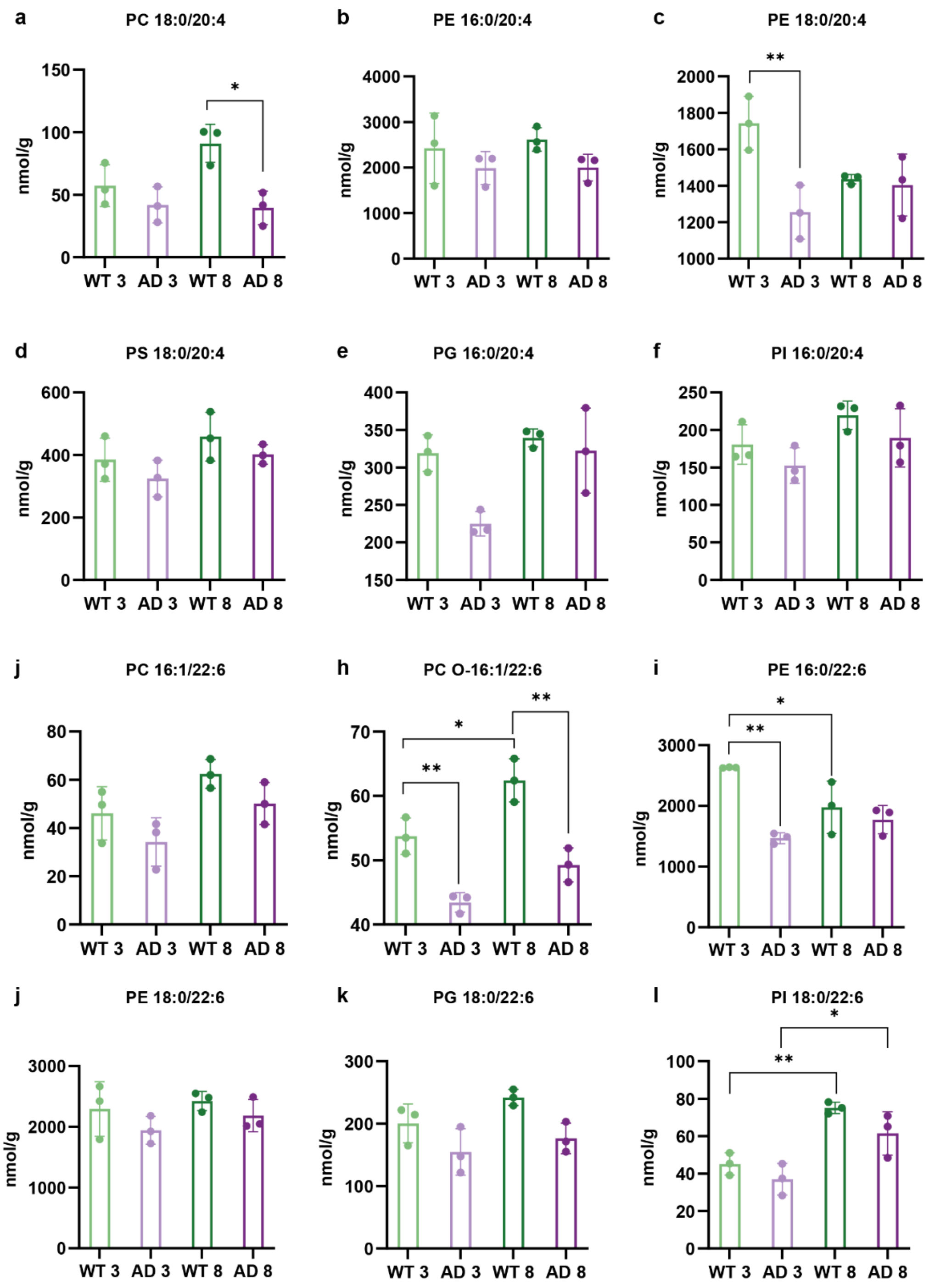

Supplementary Figure 27. Compositional variations of GPs with C20:4 and DHA at *sn*-2 position among the AD and WT groups at 3 and 8 months in the cortex. Concentrations of PC 18:0/20:4 (p (WT 8 vs. AD 8) = 0.0125) (**a**), PE 16:0/20:4 (p (WT 3 vs. AD 3) = 0.0095) (**b**), PE 18:0/20:4 (**c**), PS 18:0/20:4 (**d**), PG 16:0/20:4 (**e**), PI 16:0/20:4 (**f**), PC 16:1/22:6 (**g**), PE O-16:1/22:6 (p (WT 3 vs. AD 3) = 0.0065, p (WT 3 vs. WT 8) = 0.0173, p (AD 3 vs. AD 8)= 0.0014, p (WT 8 vs. AD 8)= 0.0403) (**h**), PE 16:0/22:6 (p (WT 3 vs. AD 3) = 0.0020, p (WT 3 vs. WT 8) =

0.0499) (**i**), PS 18:0/22:6 (**j**), PG 18:0/22:6 (**k**), PI 18:0/22:6 (*p* (WT 3 vs. WT 8) = 0.0012, *p* (AD 3 vs. AD 8) = 0.0224) (**l**) in cortex. Data are presented as mean values +/−SD (*n* = 3, **p* < 0.05, ***p* < 0.01, ****p* < 0.001 (one-way ANOVA, with correction for multiple comparisons at a 0.05 FDR)). Source data are provided as a Source Data file.

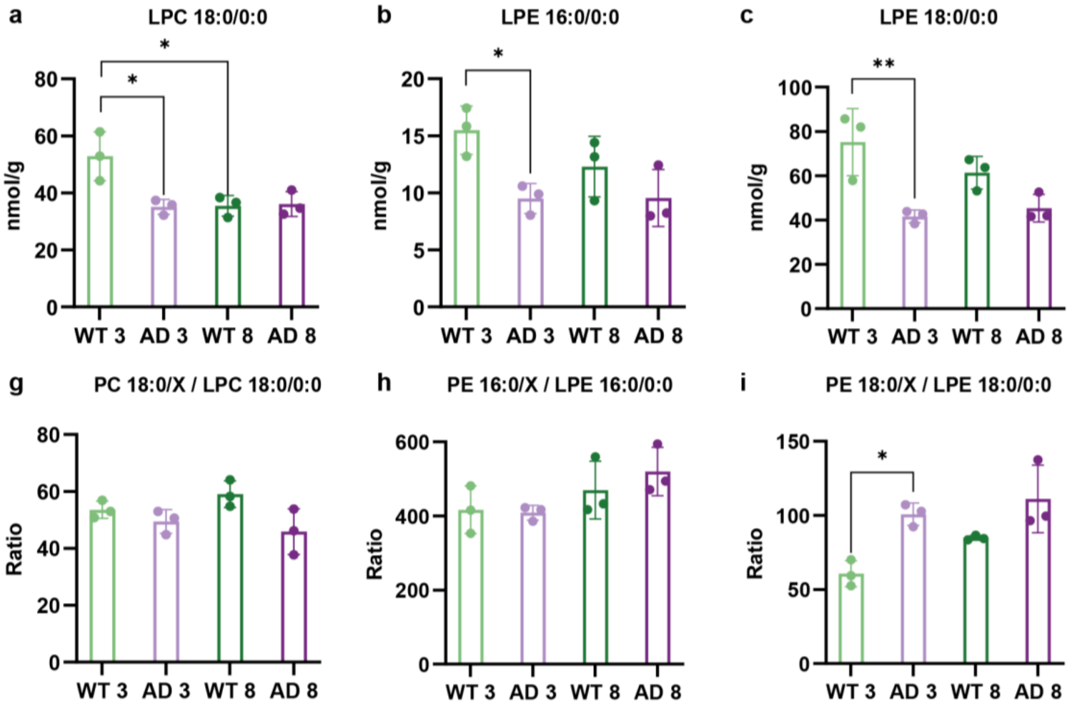

Supplementary Figure 28. Compositional variations of representative lyso-GPs and the ratio variations of representative GP/lyso-GP among the AD and WT groups at 3 and 8 months in the cortex. Concentrations of various lyso-GPs and ratios of corresponding GP/lyso-GP are presented: LPC 18:0/0:0 (*p* (WT 3 vs. AD 3) = 0.0149, *p* (WT 3 vs. WT 8) = 0.0168) (**a**), LPE 16:0/0:0 (*p* (WT 3 vs. AD 3) = 0.0430) (**b**), LPE 18:0/0:0 (*p* (WT 3 vs. AD 3) = 0.0085) (**c**), PC 18:0/X / LPC 18:0/0:0 (**d**), PE 16:0/X / LPE 16:0/0:0 (**e**), PE 18:0/X / LPE 18:0/0:0 (*p* (WT 3 vs. AD 3) = 0.0210) (**f**). Data are presented as mean values +/−SD (*n* = 3, **p* < 0.05, ***p* < 0.01, ****p* < 0.001 (one-way ANOVA, with correction for multiple comparisons at a 0.05 FDR)). Source data are provided as a Source Data file.

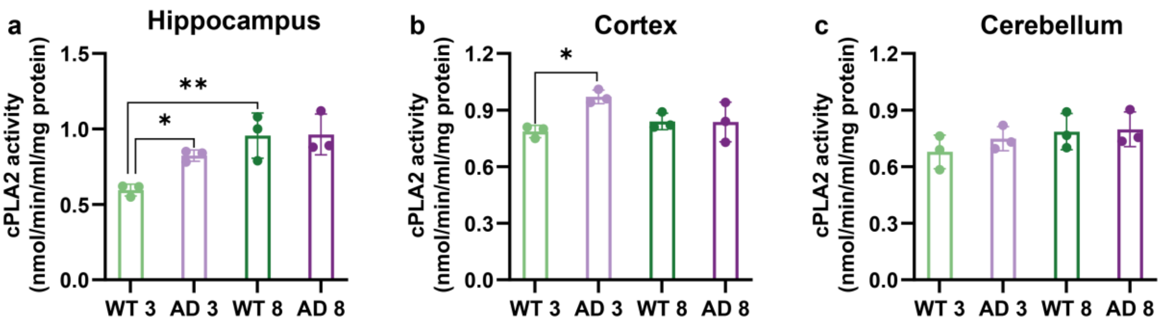

Supplementary Figure 29. cPLA2 activities in mouse brain tissues from different genotypes and ages. cPLA2 activities of hippocampus (**a**) (p (WT 3 vs. AD 3) = 0.0399, p (WT 3 vs. WT 8) = 0.0061), cortex (**b**) (p (WT 3 vs. AD 3) = 0.0349), and cerebellum (**c**) are presented. Data are presented as mean values +/−SD (n = 3, $*p < 0.05$, $**p < 0.01$, (one-way ANOVA with correction for multiple comparisons using the two-stage linear step-up procedure of Benjamin, Krieger and Yekutieli at a 0.05 FDR)). Source data are provided as a Source Data file.

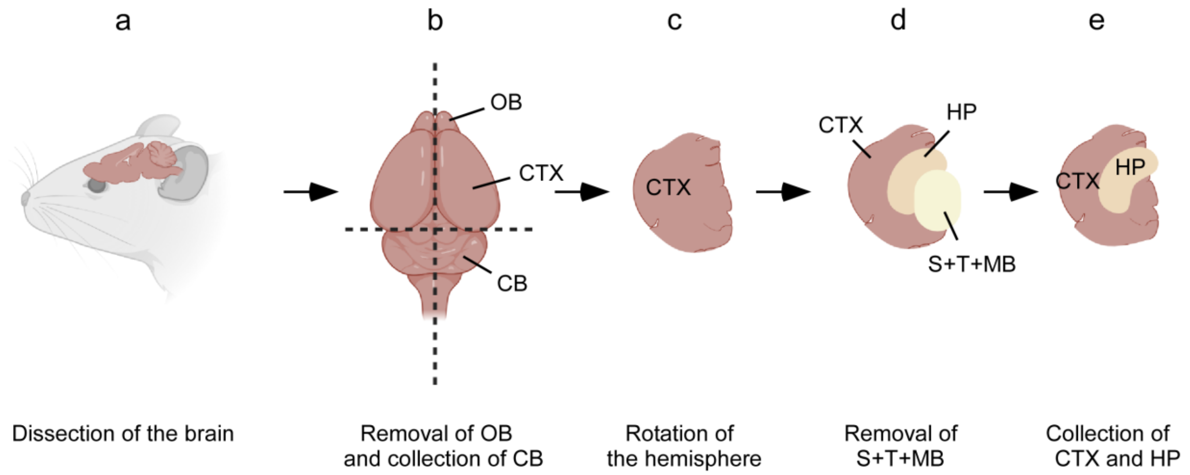

Supplementary Figure 30. Schematic representation of mouse brain dissection. The whole brain is removed from the skull (**a**) and placed on a dissection surface. Both the olfactory bulbs (OB) and the cerebellum (CB) are removed prior to sagittal separation of the two hemispheres (b). The single hemisphere is rotated to expose the medial surface (**c**) prior to removal of the striatum (S), thalamus (T) and midbrain (MB) to expose the hippocampus (**d**). The hippocampus (HP) and cortex (CTX) are then separated. Diagram was created with BioRender.com, released under a Creative Commons Attribution-NonCommercial-NoDerivs 4.0 International license.

.

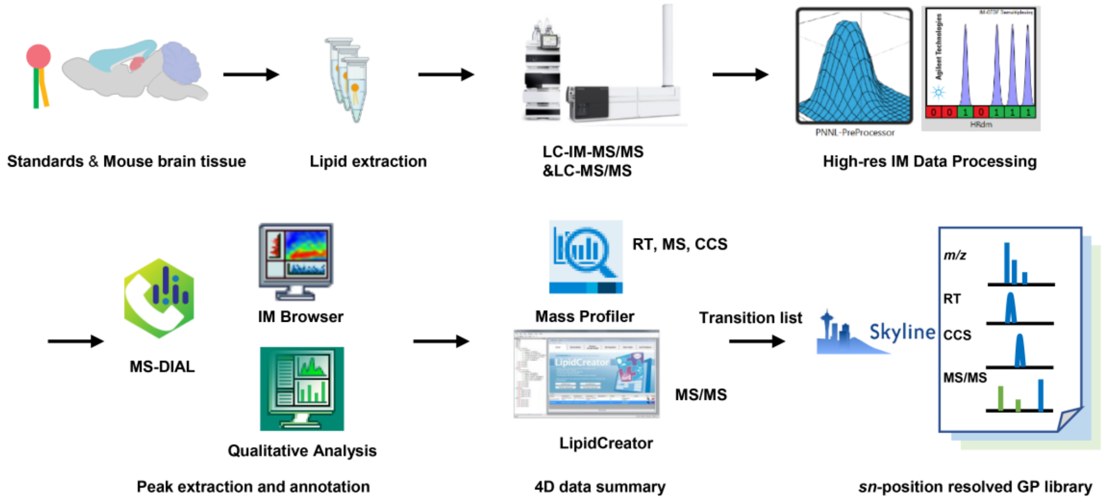

Supplementary Figure 31. **The workflow for LC-high resolution-IM-MS/MS based 4D *sn*-resolved GP library construction.** The workflow involves lipid extraction, data acquisition, the peak extraction and annotation using MS-DIAL, IM Browser and Qualitative analysis, 4D data summary and transition list generation using Mass Profiler and LipidCreator. Diagram was created with BioRender.com, released under a Creative Commons Attribution-NonCommercial-NoDerivs 4.0 International license.

Supplementary Table 1. The information on GP standards used in this study.

No.	Name	Part Number	Vender
1	PC 16:0/18:1(9Z) (IsoPure)	792453	Avanti
2	PC 16:0/18:1(11Z) (IsoPure)	792455	Avanti
3	PC 18:1(9Z)/18:1(9Z)	850375	Avanti
4	PC 18:1(11Z)/18:1(11Z)	790626	Avanti
5	PC 18:0/18:2 (9Z, 12Z)	850468	Avanti
6	PC 15:0/18:1(d7)	791637	Avanti
7	PC 16:0/22:6 (4Z,7Z,10Z,13Z,16Z,19Z)	850461	Avanti
8	PE 16:0/18:1(9Z) (IsoPure)	792518	Avanti
9	PE 16:0/18:1(9Z)	850757	Avanti
10	PE 18:1/16:0(9Z) (IsoPure)	792519	Avanti
11	PE 15:0/18:1(d7)	791638	Avanti
12	PS 16:0/18:1(9Z) (IsoPure)	792522	Avanti
13	PS 18:1/16:0(9Z) (IsoPure)	792523	Avanti
14	PS 15:0/18:1(d7)	791639	Avanti
15	PG 18:1(9Z)/18:1(9Z)	840475	Avanti
16	PG 16:0/16:0	840455	Avanti
17	PG 15:0/18:1(d7)	791640	Avanti
18	PI 18:1(9Z)/18:1(9Z)	850149	Avanti
19	PI 15:0/18:1(d7)	791641	Avanti
20	PA 18:1(9Z)/18:1(9Z)	840875	Avanti
21	PA 15:0/18: 1(d7)	791642	Avanti
23	EquiSPLASH	330731	Avanti

Supplementary Table 2. GP standards used for the comparison of quantitative performance of HRdm IM-MS with PLA2 digestion.

GP Standards	Species	PLA2	IM-MS
Non-isomer pure PE 16:0/18:1 (9Z)	PE 16:0/18:1	80.96 ± 1.82	81.28 ± 1.51
	PE 18:1/16:0	19.04 ± 1.82	18.72 ± 1.51
Isopure PE 16:0/18:1 (9Z)	PE 16:0/18:1	100.00 ± 0.00	100.00 ± 0.00
Isopure PE 18:1 (9Z)/16:0	PE 18:1/16:0	100.00 ± 0.00	100.00 ± 0.00
Non-isomer pure PC 16:0/22:6 (4Z,7Z,10Z,13Z,16Z,19Z)	PC 16:0/22:6	86.64 ± 0.44	85.23 ± 0.46
	PC 22:6/16:0	13.36 ± 0.44	14.77 ± 0.46
Isopure PC 16:0/18:1(9Z)	PC 16:0/18:1	99.13 ± 0.17	100.00 ± 0.00
	PC 18:1/16:0	0.87 ± 0.17	0.00 ± 0.00
[a]PE 16:0/18:1 (9Z) / PE 18:1(9Z)/16:0 (50:50)	PE 16:0/18:1	49.76 ± 0.21	51.58 ± 1.23
	PE 18:1/16:0	50.24 ± 0.21	48.42 ± 1.23
PE 16:0/18:1 (9Z) / PE 18:1(9Z)/16:0 (33.3:66.7)	PE 16:0/18:1	31.82 ± 0.38	30.57 ± 1.20
	PE 18:1/16:0	68.18 ± 0.38	69.43 ± 1.20
PE 16:0/18:1 (9Z) / PE 18:1(9Z)/16:0 (20:80)	PE 16:0/18:1	21.39 ± 0.43	19.89 ± 0.37
	PE 18:1/16:0	78.61 ± 0.43	80.11 ± 0.37
PE 16:0/18:1 (9Z) / PE 18:1(9Z)/16:0 (16.7:83.3)	PE 16:0/18:1	14.1 ± 0.11	17.28 ± 0.63
	PE 18:1/16:0	85.9 ± 0.11	82.72 ± 0.63
PE 16:0/18:1 (9Z) / PE 18:1(9Z)/16:0 (9.1:90.9)	PE 16:0/18:1	9.77 ± 0.26	8.31 ± 0.82
	PE 18:1/16:0	90.23 ± 0.26	91.69 ± 0.82
PE 16:0/18:1 (9Z) / PE 18:1(9Z)/16:0 (6.25:93.75)	PE 16:0/18:1	6.23 ± 0.15	6.77 ± 0.97
	PE 18:1/16:0	93.77 ± 0.15	93.23 ± 0.97

Each measurement was performed in three replicates.

[a] Isomeric mixture of isopure PE 16:0/18:1 (9Z) and PE 18:1(9Z)/16:0.

Supplementary Table 3. The list of the 35 most common fatty acids used for MDs calculation.

No.	Systematic Name	Species Shorthand	Formula	Mass
1	Decanoic acid	FA 10:0	C10H20O2	172.1463
2	Dodecanoic acid	FA 12:0	C12H24O2	200.1776
3	Tridecanoic acid	FA 13:0	C13H26O2	214.1933
4	Tetradecanoic acid	FA 14:0	C14H28O2	228.2089
5	9Z-tetradecenoic acid	FA 14:1 (9Z)	C14H26O2	226.1933
6	Pentadecanoic acid	FA 15:0	C15H30O2	242.2246
7	9Z-pentadecenoic acid	FA 15:1 (9Z)	C15H28O2	240.2089
8	Hexadecanoic acid	FA 16:0	C16H32O2	256.2402
9	9Z-hexadecenoic acid	FA 16:1 (9Z)	C16H30O2	254.2246
10	Heptadecanoic acid	FA 17:0	C17H34O2	270.2559
11	10Z-heptadecenoic acid	FA 17:1(10Z)	C17H32O2	268.2402
12	Octadecanoic acid	FA 18:0	C18H36O2	284.2715
13	9Z-octadecenoic acid	FA 18:1 (9Z)	C18H34O2	282.2559
14	9Z,12Z-octadecadienoic acid	FA 18:2 (9Z,12Z)	C18H32O2	280.2402
15	9Z,12Z,15Z-octadecatrienoic acid	FA 18:3 (9Z,12Z,15Z)	C18H30O2	278.2246
16	6Z,9Z,12Z,15Z-octadecatetraenoic acid	FA 18:4 (6Z,9Z,12Z,15Z)	C18H28O2	276.2089
17	Nonadecanoic acid	FA 19:0	C19H38O2	298.2872
18	10Z-nonadecenoic acid	FA 19:1 (10Z)	C19H36O2	296.2715
19	Eicosanoic acid	FA 20:0	C20H40O2	312.3028
20	11Z-eicosenoic acid	FA 20:1 (11Z)	C20H38O2	310.2872
21	11Z,14Z-eicosadienoic acid	FA 20:2 (11Z,14Z)	C20H36O2	308.2715
22	8Z,11Z,14Z-eicosatrienoic acid	FA 20:3 (8Z,11Z,14Z)	C20H34O2	306.2559
23	5Z,8Z,11Z,14Z-eicosatetraenoic acid	FA 20:4 (5Z,8Z,11Z,14Z)	C20H32O2	304.2402
24	5Z,8Z,11Z,14Z,17Z-eicosapentaenoic acid	FA 20:5 (5Z,8Z,11Z,14Z,17Z)	C20H30O2	302.2246
25	Heneicosanoic acid	FA 21:0	C21H42O2	326.3185
26	Docosanoic acid	FA 22:0	C22H44O2	340.3341
27	13Z-docosenoic acid	FA 22:1 (13Z)	C22H42O2	338.3185
28	13Z,16Z-docosadienoic acid	FA 22:2 (13Z,16Z)	C22H40O2	336.3028

29	10Z, 13Z,16Z-docosadienoic acid	FA 22:3 (10Z,13Z,16Z)	C22H38O2	334.2872
30	7Z,10Z,13Z,16Z-docosadienoic acid	FA 22:4 (7Z,10Z,13Z,16Z)	C22H36O2	332.2715
31	4Z,7Z,10Z,13Z,16Z-docosadienoic acid	FA 22:5 (4Z,7Z,10Z,13Z,16Z)	C22H34O2	330.2559
32	4Z,7Z,10Z,13Z,16Z,19Z-docosadienoic acid	FA 22:6 (4Z,7Z,10Z,13Z,16Z,19Z)	C22H32O2	328.2402
33	Tetracosanoic acid	FA 24:0	C24H48O2	368.3654
34	15Z-tetracosenoic acid	FA 24:1 (15Z)	C24H46O2	366.3498
35	Hexacosanoic acid	FA 26:0	C26H52O2	396.3967

Supplementary Table 4. The list of optimized molecular descriptors calculated by CDK for CCS prediction.

No.	Name	No.	Name
1	Zagreb	13	khs.sssCH
2	XLogP	14	khs.ssO
3	WPATH	15	Kier2
4	WPOL	16	HybRatio
5	WTPT.1	17	ECCEN
6	MW	18	C1SP3
7	VAdjMat	19	BCUTw.1l
8	LipinskiFailures	20	BCUTp.1h
9	nRotB	21	ATSc3
10	PetitjeanNumber	22	ATSc4
11	MDEC.22	23	nAcid
12	MDEO.22	24	nA

Note: Please refer to reference 5 for the detailed description of each molecular descriptor[5].

Supplementary Table 5. The list of optimized molecular descriptors calculated by Mordred for CCS prediction.

No.	Name	No.	Name
1	nAtom	12	ATS8se
2	ATS1d	13	ATS8p
3	ATS3d	14	AATSC1s
4	ATS5d	15	BCUTp-1l
5	ATS7d	16	SpDiam_DzZ
6	ATS8d	17	SpDiam_Dzm
7	ATS5s	18	TIC5
8	ATS4v	19	PEOE_VSA8
9	ATS5v	20	MDEC-22
10	ATS7v	21	WPath
11	ATS1se		

Note: Please refer to reference 6 for the detailed description of each molecular descriptor[6].

Supplementary Table 6. The list of molecular descriptors with over 0.1% differences between *sn*-position isomers from Mordred.

No.	Name	No.	Name	No.	Name
1	SM1_A	12	SpDiam_Dzp	23	MAXdssC
2	VR1_A	13	SpDiam_Dzi	24	MINssCH2
3	VR2_A	14	SpDiam_Dt	25	MINdsCH
4	VR3_A	15	SM1_Dt	26	MINsssCH
5	BalabanJ	16	DetourIndex	27	MINdssC
6	SpDiam_DzZ	17	SM1_D	28	ECIndex
7	SpDiam_Dzm	18	SdsCH	29	EState_VSA2
8	SpDiam_Dzv	19	SsssCH	30	EState_VSA3
9	SpDiam_Dzse	20	MAXssCH2	31	MDEC-23
10	SpDiam_Dzpe	21	MAXdsCH	32	WPath
11	SpDiam_Dzare	22	MAXsssCH		

Note: Please refer to reference 6 for the detailed description of each molecular descriptor.

Supplementary Table 7. The list of optimized 37 molecular descriptors from 253 molecular descriptors (including all 221 MDs from CDK and 32 Modred MDs which demonstrated > 0.1% difference between *sn*-position isomers) for building the prediction model.

No.	Name	No.	Name	No.	Name
1	Zagreb	14	khs.dssC	27	ALogP
2	XLogP	15	khs.dO	28	nAcid
3	WPATH	16	khs.ssO	29	SM1_A
4	WPOL	17	Kier2	30	VR1_A
5	WTPT.1	18	Kier3	31	VR2_A
6	MW	19	HybRatio	32	VR3_A
7	VAdjMat	20	ECCEN	33	SM1_D
8	nRotB	21	C1SP3	34	MAXdssC
9	MDEC.12	22	BCUTw.1l	35	MINdssC
10	MDEC.22	23	BCUTw.1h	36	EState_VSA2
11	nAtomLAC	24	BCUTc.1l	37	EState_VSA3
12	nAtomLC	25	BCUTp.1h		
13	khs.ssCH2	26	ATSc4		

MDs 1-28 were calculated by R package rcdk; MDs 29-37 were calculated by Mordred software.

Note: Please refer to references 5 and 6 for the detailed description of each molecular descriptor.

Supplementary Table 8. The list of optimized molecular descriptors calculated by CDK for retention time prediction.

No.	Name	No.	Name	No.	Name
1	Fsp3	18	nAtomLC	35	C1SP2
2	tpsaEfficiency	19	khs.ssCH2	36	C1SP3
3	XLogP	20	khs.dsCH	37	C2SP3
4	WPATH	21	khs.sNH3	38	bpol
5	WPOL	22	khs.ssssN	39	nB
6	WTPT.2	23	khs.sOH	40	BCUTw.1l
7	MW	24	khs.ssO	41	BCUTw.1h
8	LipinskiFailures	25	nHBDon	42	BCUTc.1l
9	nRotB	26	fragC	43	BCUTp.1h
10	topoShape	27	VP.3	44	nBase
11	MDEC.12	28	VP.4	45	ATSm3
12	MDEC.13	29	VP.5	46	ATSc1
13	MDEC.22	30	VP.6	47	ATSc2
14	MDEC.23	31	VP.7	48	ATSc3
15	MDEO.11	32	SPC.5	49	ATSc4
16	MDEO.12	33	SC.4	50	ALogp2
17	MDEO.22	34	VC.4	51	AMR

Note: Please refer to reference 5 for the detailed description of each molecular descriptor.

Supplementary Methods

TIMS-TOF flex analysis

The TIMS data was acquired using a TIMS-TOF flex operating in electrospray ionization mode (Bruker Daltonics, Germany) as reported with a light modification[7]. PASEF-DDA scan mode was applied to obtain ions with m/z from 100 Da to 1350 Da. The acquisition cycle consisted of 0.1 s with the mobility scan range of mobility from 0.55 to 1.86 $V \cdot s/cm^2$. IM-MS parameters were set as follows: capillary voltages were +4500 V for positive ionization mode; nebulizer pressure, 2.5 bar; dry gas, 6.0 L/min; dry temperature, 200 °C; ramping time, 100 to 1000 ms; collision energy, 35 eV in positive ion mode. Both the TIMS and mass calibration of the instrument was carried out from the Agilent tuning mix before sample runs. The mobilograms were processed and integrated using DataAnalysis (version 6.1, Bruker)

Cyclic ion mobility (cIM)-MS analysis

IM-MS data were acquired in positive mode on a SELECT SERIES Cyclic IMS (Waters, Wilmslow, U.K.). Approximately 5 µL of each sample was loaded into a borosilicate glass needle and placed into a custom-built nanospray ion source as described previously[8]. The mass range was set to *m/z* 50–1200. The essential parameters were as follows: capillary voltage 1.2 kV, sampling cone 60 V, source offset 30 V, source temperature 120 °C. The cIM device was set to a gas pressure of 1.7 mbar, a traveling wave height of 22 V, and a velocity of 375 m/s. Data are processed using Masslynx (version 4.1).

References

1. Maccarone, A. T., *et al.* Characterization of acyl chain position in unsaturated phosphatidylcholines using differential mobility-mass spectrometry. *J. Lipid Res.* **55**, 1668-1677 (2014).
2. Butler, K. E., *et al.* High-Resolution Demultiplexing (HRdm) Ion Mobility Spectrometry-Mass Spectrometry for Aspartic and Isoaspartic Acid Determination and Screening. *Anal. Chem.* **94**, 6191-6199 (2022).
3. Kyle, J. E., *et al.* Uncovering biologically significant lipid isomers with liquid chromatography, ion mobility spectrometry and mass spectrometry. *Analyst* **141**, 1649-1659 (2016).
4. May, J. C., *et al.* Resolution of Isomeric Mixtures in Ion Mobility Using a Combined Demultiplexing and Peak Deconvolution Technique. *Anal. Chem.* **92**, 9482-9492 (2020).
5. Guha, R. Chemical Informatics Functionality in R. *Journal of Statistical Software* **18**, 1-16 (2007).
6. Moriwaki, H., *et al.* Mordred: a molecular descriptor calculator. *J. Cheminformatics* **10**, 4 (2018).
7. Lerner, R., *et al.* Four-dimensional trapped ion mobility spectrometry lipidomics for high throughput clinical profiling of human blood samples. *Nat. Commun.* **14**, 937 (2023).
8. Li, G., DeLaney, K., Li, L. Molecular basis for chirality-regulated Abeta self-assembly and receptor recognition revealed by ion mobility-mass spectrometry. *Nat. Commun.* **10**, 5038 (2019).
