# Supplementary material for: Spatially and temporally probing distinctive glycerophospholipid alterations in Alzheimer’s disease mouse brain via high-resolution ion mobility-enabled sn-position resolved lipidomics
Source: Nat Commun. 2024 Jul 24;15:6252. doi: 10.1038/s41467-024-50299-9 (PMC11269705; doi:10.1038/s41467-024-50299-9)
Supplement: Supplementary file 2 — Reporting Summary [file 41467_2024_50299_MOESM2_ESM.pdf]

nature portfolio

Corresponding author(s): Lingjun Li

Last updated by author(s): Jun 22, 2024

Reporting Summary

Nature Portfolio wishes to improve the reproducibility of the work that we publish. This form provides structure for consistency and transparency in reporting. For further information on Nature Portfolio policies, see our Editorial Policies and the Editorial Policy Checklist.

Statistics

For all statistical analyses, confirm that the following items are present in the figure legend, table legend, main text, or Methods section.

n/a	Confirmed	
☐	☒	The exact sample size (*n*) for each experimental group/condition, given as a discrete number and unit of measurement
☐	☒	A statement on whether measurements were taken from distinct samples or whether the same sample was measured repeatedly
☐	☒	The statistical test(s) used AND whether they are one- or two-sided<br>*Only common tests should be described solely by name; describe more complex techniques in the Methods section.*
☐	☒	A description of all covariates tested
☐	☒	A description of any assumptions or corrections, such as tests of normality and adjustment for multiple comparisons
☐	☒	A full description of the statistical parameters including central tendency (e.g. means) or other basic estimates (e.g. regression coefficient) AND variation (e.g. standard deviation) or associated estimates of uncertainty (e.g. confidence intervals)
☐	☒	For null hypothesis testing, the test statistic (e.g. *F*, *t*, *r*) with confidence intervals, effect sizes, degrees of freedom and *P* value noted<br>*Give P values as exact values whenever suitable.*
☒	☐	For Bayesian analysis, information on the choice of priors and Markov chain Monte Carlo settings
☐	☒	For hierarchical and complex designs, identification of the appropriate level for tests and full reporting of outcomes
☒	☐	Estimates of effect sizes (e.g. Cohen's *d*, Pearson's *r*), indicating how they were calculated

Our web collection on statistics for biologists contains articles on many of the points above.

Software and code

Policy information about availability of computer code

Data collection	LC-IM-MS data acquisitions were carried out using Mass Hunter Workstation (ver 11.0, Agilent)
Data analysis	High-resolution demultiplexing (HRdm) and CCS determination were performed with PNNL PreProcessor (ver 4.0 build 2022.02.17, https://omics.pnl.gov/software/pnnl-preprocessor), MassHunter IM-MS Browser (ver10.0, Agilent) and High-Resolution Demultiplexer (ver 2.0, Agilent). MSDIAL (Version 4.80) was used for lipid identification. Experimental four-dimensional library and extended libraries were constructed by Skyline-daily (ver 22.2.1, MacCoss Lab Software). Molecular descriptors (MDs) were obtained by using R (ver 4.2.1) and Mordred (https://mordred.phs.osaka-u.ac.jp). Statistical analysis were performed with R (ver 4.2.1), Origin 2020, and GraphPad Prism 9.0.

For manuscripts utilizing custom algorithms or software that are central to the research but not yet described in published literature, software must be made available to editors and reviewers. We strongly encourage code deposition in a community repository (e.g. GitHub). See the Nature Portfolio guidelines for submitting code & software for further information.

Data

Policy information about availability of data

All manuscripts must include a data availability statement. This statement should provide the following information, where applicable:

- Accession codes, unique identifiers, or web links for publicly available datasets
- A description of any restrictions on data availability
- For clinical datasets or third party data, please ensure that the statement adheres to our policy

The data that support the findings of this study and all curated libraries are available in the supplementary material of this article. The information of 498 GPs in the

experimental GP library and 2500 GPs in the extended GP library are provided in Supplementary Data 4 and 7, respectively. The internal validation and external validation results of CCS and retention time are provided in Supplementary Data 5 and 6. The identified GPs in the mouse brain and their abundances are provided in Supplementary Data 8 and 10, respectively. All source code for CCS prediction is provided in Github (https://github.com/lingjunli-research/Glycerophospholipid-CCS) and Zenodo (https://doi.org/10.5281/zenodo.12351060). The raw data generated in this study has been deposited in Zenodo (https://doi.org/10.5281/zenodo.12327665). Source data are provided with this paper.

Research involving human participants, their data, or biological material

Policy information about studies with human participants or human data. See also policy information about sex, gender (identity/presentation), and sexual orientation and race, ethnicity and racism.

Reporting on sex and gender	N/A. Sex and gender based analysis is not relevant for the study.
Reporting on race, ethnicity, or other socially relevant groupings	N/A. Analysis of race, ethnicity, and other social groupings are not relevant for this study.
Population characteristics	N/A
Recruitment	N/A
Ethics oversight	Animal experiments were performed in accordance with the National Institutes of Health Guide for the Care and Use of Laboratory Animals and were approved by the Institutional Animal Care and Use Committee of the University of Wisconsin-Madison (protocol #M005120).

Note that full information on the approval of the study protocol must also be provided in the manuscript.

Field-specific reporting

Please select the one below that is the best fit for your research. If you are not sure, read the appropriate sections before making your selection.

☒ Life sciences ☐ Behavioural & social sciences ☐ Ecological, evolutionary & environmental sciences

For a reference copy of the document with all sections, see nature.com/documents/nr-reporting-summary-flat.pdf

Life sciences study design

All studies must disclose on these points even when the disclosure is negative.

Sample size	The biological sample size ≥3 were chosen to support statistical analysis and variation expected based on previous experience. All experiments were performed at least three biological replicates. The exact sample size for each experiments is reported in the relevant figure legend. Multiple tests and analyses were performed as described in the manuscript to ensure that the samples are representative and the results are conclusive.
Data exclusions	No data was excluded from the analyses.
Replication	The number of replicates for each specific experiment is indicated throughout the manuscript text, figure legends and methods. All attempts of replication were successful.
Randomization	All mice were allocated randomly.
Blinding	Blinding during group allocations was not adopted during data collection since we need to determine which group has significant changes compared to the control groups. The data analysis was performed in an objective and unbiased fashion.

Reporting for specific materials, systems and methods

We require information from authors about some types of materials, experimental systems and methods used in many studies. Here, indicate whether each material, system or method listed is relevant to your study. If you are not sure if a list item applies to your research, read the appropriate section before selecting a response.

Materials & experimental systems

n/a	Involved in the study
x	Antibodies
x	Eukaryotic cell lines
x	Palaeontology and archaeology
	x Animals and other organisms
x	Clinical data
x	Dual use research of concern
x	Plants

Methods

n/a	Involved in the study
x	ChIP-seq
x	Flow cytometry
x	MRI-based neuroimaging

Animals and other research organisms

Policy information about studies involving animals; ARRIVE guidelines recommended for reporting animal research, and Sex and Gender in Research

Laboratory animals	APP695/swe/PS1-dE9 (APP/PS1) double transgenic male mice were obtained from Jackson Laboratory (MMRRC Stock No. 34832-JAX). Genotyping from tail DNA was performed at weaning by Transnetyx (Cordova, TN). APP/PS1 mice were studied at the age of three months (n = 3) and eight months (n = 3). Male mice were studied with wild-type (WT) littermates used as controls. WT mice were also studied at the age of three months (n = 3) and eight months (n = 3). All male mice were housed in standard cages provided by the University Laboratory Animal Resources and grouped with littermates with 1–5 mice per cage. Sex and gender-based analysis is not considered in this the study. Animals were housed in facilities with a standard 12-hour light-dark cycle, humidity of 50% at 24 ° C, and were provided standard chow and water ad libitum.
Wild animals	This study did not involve wild animals.
Reporting on sex	The mice used in this project are male. Our study is not relevant to sex and gender differences.
Field-collected samples	No field-collected samples were used in this study.
Ethics oversight	Animal experiments were performed in accordance with the National Institutes of Health Guide for the Care and Use of Laboratory Animals and were approved by the Institutional Animal Care and Use Committee of the University of Wisconsin-Madison (protocol #M005120).

Note that full information on the approval of the study protocol must also be provided in the manuscript.
