# Supplementary material for: Spatially and temporally probing distinctive glycerophospholipid alterations in Alzheimer’s disease mouse brain via high-resolution ion mobility-enabled sn-position resolved lipidomics
Source: Nat Commun. 2024 Jul 24;15:6252. doi: 10.1038/s41467-024-50299-9 (PMC11269705; doi:10.1038/s41467-024-50299-9)
Supplement: Supplementary file 3 — Description of Additional Supplementary Files [file 41467_2024_50299_MOESM3_ESM.pdf]

Description of Additional Supplementary Files

Files File Name: Supplementary Data 1

Description: The Z-matrix (Ångströms) corresponding to the energy-minimized structure of the GP *sn*-position isomer pairs.

Files File Name: Supplementary Data 2

Description: GP species identified at the fatty acyl composition level in pooled mouse brain by LC-MS/MS (QTOF-only mode).

Files File Name: Supplementary Data 3

Description: GP species identified at the *sn*-position resolved level in pooled mouse brain by LC-HRdm-IM-MS/MS.

Files File Name: Supplementary Data 4

Description: The 4D information of 498 GPs in the experimental GP library.

Files File Name: Supplementary Data 5

Description: Internal validations of predicted CCS values by using optimized MDs from CDK, Mordred, MDs selected from CDK and Mordred; external validation of predicted CCS values.

Files File Name: Supplementary Data 6

Description: The internal and external validations of predicted retention times by using optimized MDs from CDK.

Files File Name: Supplementary Data 7

Description: The 4D information of 2500 GPs in the extended GP library.

Files File Name: Supplementary Data 8

Description: List of identified GPs with *sn*-position resolution in mouse brain.

Files File Name: Supplementary Data 9

Description: Information on GPs in clusters 1-5 for HCA-based discrimination between the AD and WT groups in three functional mouse brain regions of mice.

Files File Name: Supplementary Data 10

The abundance of GPs in three brain regions from mice between AD and WT at ages of 3 and 8 months.

Files File Name: Supplementary Data 11

Description: Information on GPs in clusters 1-5 for HCA-based discrimination of the hippocampus in AD and WT groups at the ages of 3 and 8 months.

Files File Name: Supplementary Data 12

Description: Information on GPs with significant changes in different brain regions of AD and aging mice.
